# In vivo tibialis anterior muscle mechanics through force estimation using ankle joint moment and shear wave elastography

**DOI:** 10.1038/s41598-025-18292-4

**Published:** 2025-09-12

**Authors:** Cemre Su Kaya Keles, Jennifer Hiller, Manuela Zimmer, Filiz Ates

**Affiliations:** Institute of Structural Mechanics and Dynamics in Aerospace Engineering, University of Stuttgart, Stuttgart, Germany

**Keywords:** Muscle architecture, Muscle force estimation, Muscle mechanics, Shear wave elastography, Skeletal muscle stiffness, Tibialis anterior, Biotechnology, Physiology, Medical research, Engineering

## Abstract

Understanding how individual muscles contribute to joint mechanics is crucial for biomechanics. This study investigated the tibialis anterior (TA) shear modulus using shear wave elastography (SWE) and studied its relationship with ankle angle, contraction intensity, and joint moment-derived TA force and stress. Fourteen healthy volunteers (seven females, 26.43 ± 3.67 years) participated. SWE from TA, EMG, and ankle joint moment data were collected across ankle angles (− 15° dorsiflexion to 45° plantar flexion) during rest, maximum voluntary contraction (MVC), and isometric submaximal contractions. TA muscle length, passive ankle joint moment, and TA passive shear modulus increased with increasing plantar flexion (*p* < 0.001). During MVC, ankle joint moment peaked at 15° (50.13 Nm ± 15.54 Nm) whereas shear modulus remained unchanged (122.96 ± 9.87 kPa) across muscle lengths (*p* = 0.068). SWE reflected contractions at 25%, 50%, and 75% MVC (*p* < 0.001). TA force estimates peaked between 15° and 30°, with no significant decrease beyond this range. While SWE captured length-dependent passive properties and contraction intensity changes, the shear modulus at MVC (a stiffness measure obtained from SWE) did not align with the tangent modulus (derived from joint-moment-based force–length characteristics). Emphasizing the need for validation, SWE could serve as a valuable tool for understanding muscle mechanics and muscles’ roles in joint dynamics.

**Supplementary Information:**

The online version contains supplementary material available at 10.1038/s41598-025-18292-4.

## Introduction

Understanding in vivo muscle mechanics is essential for defining muscle function and its potential impact on joint function, as well as the adaptations of muscles during training, aging, or disease. While individual muscle forces can be estimated from measured joint moments, this approach has substantial limitations. Joint moments represent the combined effects of all synergistic and antagonistic muscles crossing a joint, each with a unique architecture, geometry, and moment arm^1^. Key parameters, such as muscle length and cross-sectional area, fascicle length, and pennation angle, influence muscle force and joint moment production^2^. Accurate representation of these parameters is fundamental for biomechanical and musculoskeletal modeling, and developing targeted interventions in sports and clinical settings. However, these calculations often rely on anthropometric assumptions that may not reflect individual muscle function, introducing potential inaccuracies^3^.

Intraoperative force measurements from tendons provide direct experimental information on in vivo muscle force characteristics. Despite being utilized by only a few research groups, this approach has significantly enhanced our understanding of muscle mechanics in healthy individuals^4,5^ and patient populations^6–11^. However, its invasive nature limits its feasibility for many healthy muscles and for quantifying voluntary activities.

Simulating human movement offers an alternative to studying muscle force-generating behavior. Inverse dynamics, a common approach, estimates muscle forces based on their moment-generation capacities at joints^12^. However, these models’ assumptions (e.g., a simplified representation of musculoskeletal geometry, optimum muscle lengths, and maximal force production capacities) may limit their accuracy, particularly in capturing inter-individual variability^13^. Surface electromyography (EMG) can also complement these models by estimating muscle forces^14^, yet it cannot capture the passive state of muscles.

Shear wave elastography (SWE) has emerged as a promising non-invasive technique for evaluating local muscle properties. This method uses a specially designed ultrasound transducer to emit focused energy beams into the tissue, generating acoustic radiation forces within the muscle. This, in turn, results in the formation of shear waves that propagate perpendicular to the transducer transmission direction^15^. The transducer captures these waves at ultrafast frame rates, enabling the measurement of the shear wave velocity (SWV) and the calculation of the shear modulus. The shear modulus was shown to correlate with muscle forces^16–18^. For linear elastic materials, stiffness is defined as the ratio of shear stress to shear strain. Similar to the length dependence of muscle force, the shear modulus also exhibits length-dependent behavior, both in a passive state and during contraction^19–22^.

Several studies have demonstrated that shear modulus, an index of muscle stiffness, can be used to predict joint moments and passive and active muscle forces^19,23–27^. Therefore, SWE holds considerable potential for estimating muscle force production and force distribution among different muscles crossing the same joint. However, both material properties and muscle stress are known to affect SWE output^28^. Additionally, recent research indicates that factors such as pennation angle^29–31^ and muscle pre-tension^31^ influence SWV. These findings underscore the need for further research to refine SWE application in characterizing in vivo muscle properties.

Research on dorsiflexors, such as the tibialis anterior (TA) muscle, is limited, with few studies addressing the length-dependency of passive and active shear moduli^15,19^. TA muscle plays a critical role in joint movement and overall foot function as the primary facilitator of dorsiflexion (DF)^32,33^. It is essential for maintaining balance and controlling foot positioning^34^. Weakness or dysfunction in the TA can lead to considerable mobility issues, such as foot drop^35,36^, highlighting its importance in both functional and clinical contexts.

In this study, we aimed to assess the in vivo mechanical properties of the TA muscle under both passive and active conditions. We hypothesized that (i) the shear modulus of the TA muscle increases as the muscle lengthens (i.e., from DF to plantar flexion (PF) joint positions) in the passive state and (ii) it reflects different contraction intensities in the active state. Additionally, we computed joint moment-driven in vivo muscle force–length and stress–strain relationships based on muscle architectural parameters within the superficial and deep compartments of the TA muscle. These results were compared with those obtained using the SWE approach to further enhance our understanding of in vivo muscle mechanics.

## Results

### Anthropometrics and muscular architecture

The study involved 14 participants with a body weight of 73.71 ± 11.28 kg, body height of 174.79 ± 8.90 cm, and body mass index of 24.09 ± 2.96 kg/m^2^; all data given as mean ± standard deviation. The tibia length and calf circumference were 39.37 ± 2.76 and 38.14 ± 2.82 cm, respectively. An ankle angle of 0° was defined as the position where the shank and sole were perpendicular. Negative ankle angles represented DF, while positive angles indicated PF. The neutral ankle angle in the supine position (40.07 ± 9.47°) ranged from PF at 25° to 56°. Active ankle range of motion (ROM) was 74.14 ± 9.00° (ranging from − 8.29 ± 5.21° to 65.86 ± 10.77°), while passive ankle ROM was 92.93 ± 7.95° (ranging from − 18.29 ± 4.08° to 74.64 ± 9.38°). All participants were able to position their ankles in all tested positions. For those with an active DF limit slightly below 15°, the 15° DF position could be reached with a slight push without causing discomfort.

Ankle angle significantly affected the TA muscle length (F(4, 52) = 122.79, *p* < 0.001) but had no significant effect on its tendon length (F(4, 52) = 1.99, *p* = 0.110) (Table 1). Post hoc analysis showed significant differences in muscle length across all pairwise comparisons (*p* ≤ 0.001 for all comparisons except between − 15° and 0°, *p* = 0.035). Muscle length increased from DF to PF positions. The maximum muscle length measured at 45° (26.48 ± 1.58 cm) was 15.88% longer compared to the minimum measured at − 15° (22.86 ± 1.49 cm).

**Table 1:** Anthropometrics of the tibialis anterior (TA) muscle.

	Ankle angle				
	− 15°	0°	15°	30°	45°
Muscle length (cm) ^+^	22.86 ± 1.49	23.66 ± 1.72	24.41 ± 1.60	25.61 ± 1.56	26.48 ± 1.58
Tendon length (cm)	19.33 ± 1.52	19.47 ± 1.83	19.51 ± 1.69	19.14 ± 1.75	18.98 ± 1.91
$ACSA_{TA}$(cm^2^)	8.54 ± 2.03	7.94 ± 1.94 *	7.52 ± 1.70 *	7.09 ± 1.36 *	6.90 ± 1.36 *^,^ **
$PCSA_{TA\;superficial}$(cm^2^)	8.68 ± 1.98	7.52 ± 1.44	6.69 ± 1.76 *	6.13 ± 1.52 *^,^ **	5.45 ± 1.76 *^,^ **^,^^○,^^□^
$PCSA_{TA\;deep}$(cm^2^)	9.10 ± 1.77	7.67 ± 2.22 *	6.98 ± 1.47 *	5.68 ± 1.55 *^,^ **^,^^○^	4.34 ± 0.99 *^,^ **^,^^○,^^□^

Values are mean ± standard deviation. ACSA: Anatomical cross-sectional area; PCSA, physiological cross-sectional area. + denotes that the muscle length values for each ankle angle are significantly different in all pairwise comparisons. ^*^, ^**^, ^○^, and ^□^ indicate significant differences from the values measured at − 15°, 0°, 15°, and 30° ankle angles, respectively. *p* < 0.05 for all pairwise comparisons.

The anatomical cross-sectional area (ACSA) of the TA was significantly affected by the ankle angle (Table 1) (F(4, 52) = 18.00, *p* < 0.001). Compared to the ACSA at the shortest TA length (at − 15°), the ACSA at the longest TA length (at 45°) was 17.96% smaller (*p* < 0.001). The estimated volumes of the TA muscle and its superficial and deep compartments were 95.84 ± 25.14 cm^3^, 49.31 ± 15.33 cm^3^, and 46.53 ± 10.92 cm^3^, respectively. Detailed results on pennation angles and fascicle lengths for the superficial and deep compartments of the TA, which were used to calculate the physiological cross-sectional area (PCSA), can be found in Supplement 1. The PCSA was significantly affected by ankle angle (F(4, 52) = 93.14, *p* < 0.001) with a significant interaction effect (F(4, 52) = 2.93, *p* = 0.029), but showed no difference between muscle compartments (F(1, 13) = 0.17, *p* = 0.687), decreasing from DF to PF (Table 1).

### Passive muscle characteristics

Of the 840 EMG signals collected at rest, 64 were excluded due to EMG amplitude exceeding 5%. The passive EMG amplitudes for each muscle, averaged across ankle angles, were: TA: 1.24 ± 0.15%; EDL: 1.84 ± 0.19%; GM: 2.97 ± 0.22%; GL: 1.93 ± 0.16%; SOL: 2.55 ± 0.06%; PERL: 2.24 ± 0.09% of the EMG amplitude at maximum voluntary contraction (MVC).

**Fig. 1:**
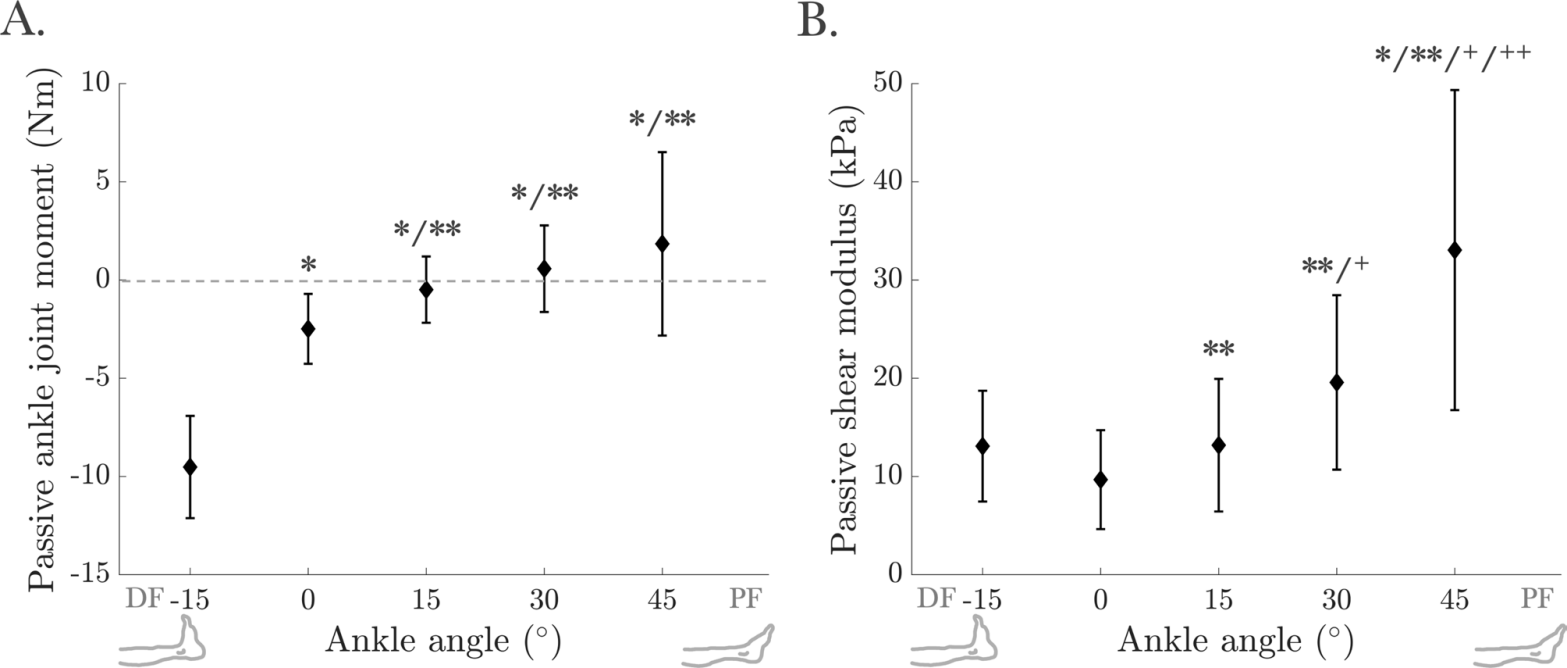
(**A**) Passive ankle joint moment and (**B**) Passive shear modulus of the tibialis anterior (TA) at rest. *, **, + , and +  + indicate significant differences from the values measured at − 15°, 0°, 15°, and 30° ankle angles, respectively.

The passive ankle joint moment increased from DF towards PF (F(4, 52) = 48.55, *p* < 0.001), with the minimum (− 9.52 ± 2.60 Nm) at − 15° and the maximum (1.84 ± 4.67 Nm) at 45° (Fig. 1A). Post hoc analysis revealed significant differences between − 15° and all other ankle angles (*p* < 0.001 for all), and between 0° and 15°/30°/45° (*p* = 0.002, *p* = 0.001, and *p* = 0.033, respectively).

The TA’s passive shear modulus increased towards PF (F(4, 52) = 27.27, *p* < 0.001), with the minimum at 0° (9.67 kPa ± 5.03 kPa) and the maximum at 45° (33.05 kPa ± 16.29 kPa) ankle angles (Fig. 1B). Post hoc analysis indicated significant differences between − 15° and 45° (*p* = 0.008), 0° and 15°/30°/45° (*p* = 0.007, *p* < 0.001, and *p* < 0.001, respectively), 15° and 30°/45° (*p* < 0.001 for both), and 30° and 45° (*p* = 0.001) ankle angles.

### Active muscle characteristics

**Fig. 2:**
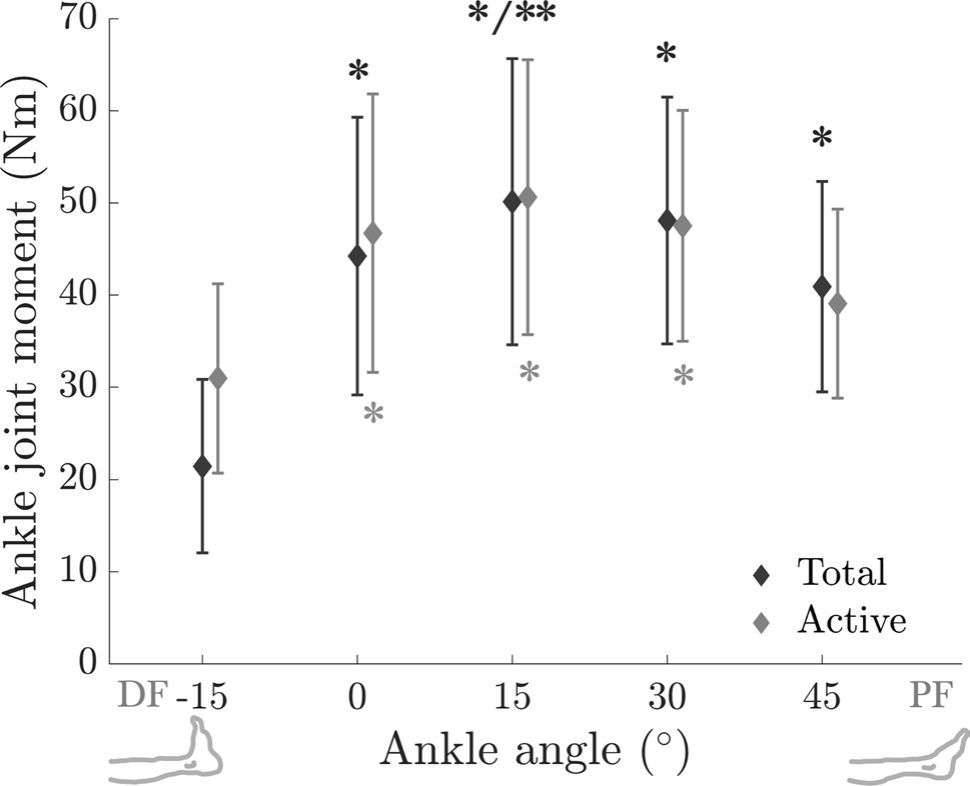
Total and active ankle joint moments at maximum voluntary contraction (MVC). *, and ** indicate significant differences from the values measured at − 15° and 0° ankle angles, respectively, with color coding distinguishing the two moments.

*Maximum voluntary contraction* Fig. 2 shows the total and active ankle joint moments at MVC, both significantly affected by the ankle angle (Total: F(4, 52) = 36.02, *p* < 0.001, and Active: F(4, 52) = 17.84, *p* < 0.001). The total ankle moment was lowest at − 15° (21.44 Nm ± 9.39 Nm) and peaked at 15° (50.13 Nm ± 15.54 Nm). Post hoc analysis indicated significant differences between − 15° and all other ankle angles (*p* ≤ 0.001 for all), and 0° and 15° (*p* = 0.006). The active ankle moment was lowest at − 15° (30.97 Nm ± 10.25 Nm) and peaked at 15° (50.62 Nm ± 14.92 Nm). Post hoc analysis indicated significant differences between − 15° and 0°/15°/30° (*p* < 0.001 for all).

**Fig. 3:**
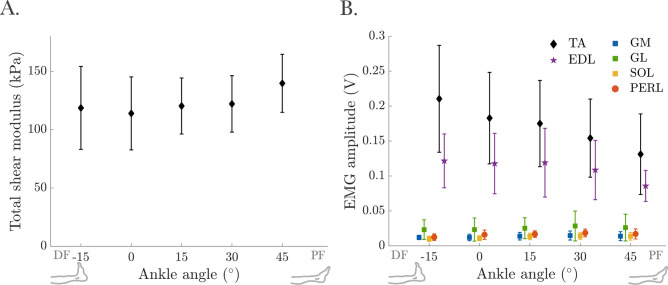
(**A**) Total shear modulus of the tibialis anterior (TA) and (**B**) EMG amplitude of tested muscles at maximum voluntary contraction (MVC).

Of the 210 SWE trials collected during MVC, 6 were excluded due to yielding one or fewer valid frames (i.e., frames with less than 75% colored pixels). The included frames contained 98.10 ± 4.23% colored pixels. 20.97 ± 18.61% of pixels reached the maximum shear modulus value. No significant effect of ankle angle was observed for the total shear modulus of the TA (F(4, 52) = 2.33, *p* = 0.068). The average total shear modulus, e.g., at 0° was 113.97 ± 31.31, and 122.96 ± 9.87 kPa across all ankle angles (Fig. 3A).

The EMG amplitudes of TA at MVC were significantly affected by the ankle angle (F(4, 52) = 19.68, *p* < 0.001) (Fig. 3B). Significant differences were observed between − 15° and 0°/15°/30°/45° (*p* = 0.003, *p* = 0.001, *p* = 0.001, and *p* = 0.002, respectively), 0° and 30°/45° (*p* = 0.047 and *p* = 0.020, respectively), and 15° and 45° (*p* = 0.022). The magnitude of differences ranged from 36.69% (− 15° vs. 45°) to 12.98% (− 15° vs. 0°). For additional EMG results, see Supplement 2.

**Fig. 4:**
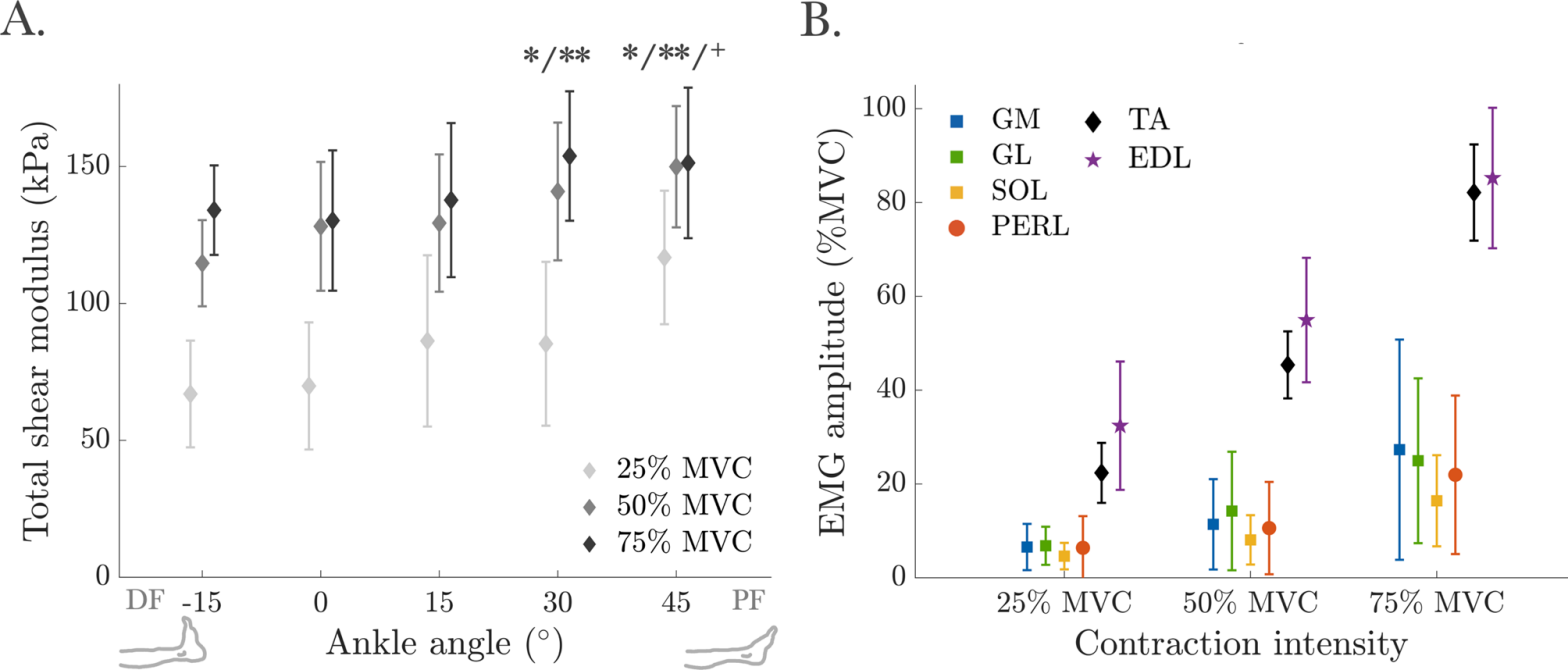
Total shear modulus of the tibialis anterior (TA) and (**B**) EMG amplitude of tested muscles during submaximal ramp contractions (25%, 50%, and 75% of maximum voluntary contraction (MVC) moment). In (**A**), *, **, and + indicate significant differences from the values measured at − 15°, 0°, and 15° ankle angles, respectively. The data in (**B**) are presented for a 0° ankle angle as a representative example, showing how the muscles’ EMG amplitudes vary with different contraction intensities (see Supplement 2).

*Submaximal ramp contractions* All SWE trials collected during the ramp contractions were included, with 99.38 ± 1.89% valid pixels. For 25%, 50%, and 75% MVC, the percentages of pixels reaching the maximum shear modulus value were 2.31 ± 4.47%, 17.01 ± 15.48%, and 24.50 ± 18.48%, respectively. The total shear modulus (Fig. 4A) was significantly affected by both the ankle angle (F(4, 52) = 19.84, *p* < 0.001) and contraction intensity (F(2, 26) = 43.91, *p* < 0.001), and a significant interaction (F(8, 104) = 3.25, *p* = 0.002) was observed. Post hoc analysis indicated significant differences between − 15° and 30°/45°, 0° and 30°/45°, and 15° and 45° (*p* ≤ 0.009 for all). Significant differences were found at each contraction intensity (*p* < 0.001 for 25% MVC and 50%/75% MVC and *p* = 0.018 for 50% and 75% MVC). Differences between 25 and 50% MVC and 25% and 75% MVC were significant at all ankle angles (*p* ≤ 0.017), while the difference between 50 and 75% MVC was significant only at − 15° (*p* = 0.025). At 25% MVC, post hoc analysis revealed significant differences between − 15° and 45° (*p* < 0.001), 0° and 45° (*p* < 0.001), and 30° and 45° (*p* = 0.016). At 50% MVC, significant differences were found between − 15° and 30°/45° (*p* = 0.041 and *p* = 0.001, respectively), and between 0° and 45° (*p* = 0.014). At 75% MVC, a significant difference was found between 0° and 30° (*p* = 0.020).

Both contraction intensity (F(2, 26) = 501.89, *p* < 0.001) and ankle angle (F(4, 52) = 26.54, *p* < 0.001) significantly affected the TA EMG amplitudes, with a significant interaction (F(8, 104) = 5.45, *p* < 0.001). TA EMG amplitudes differed significantly between all contraction intensities (Fig. 4B) at all ankle angles (Supplement 2).

### Tibialis anterior force–length and stress–strain characteristics

**Fig. 5:**
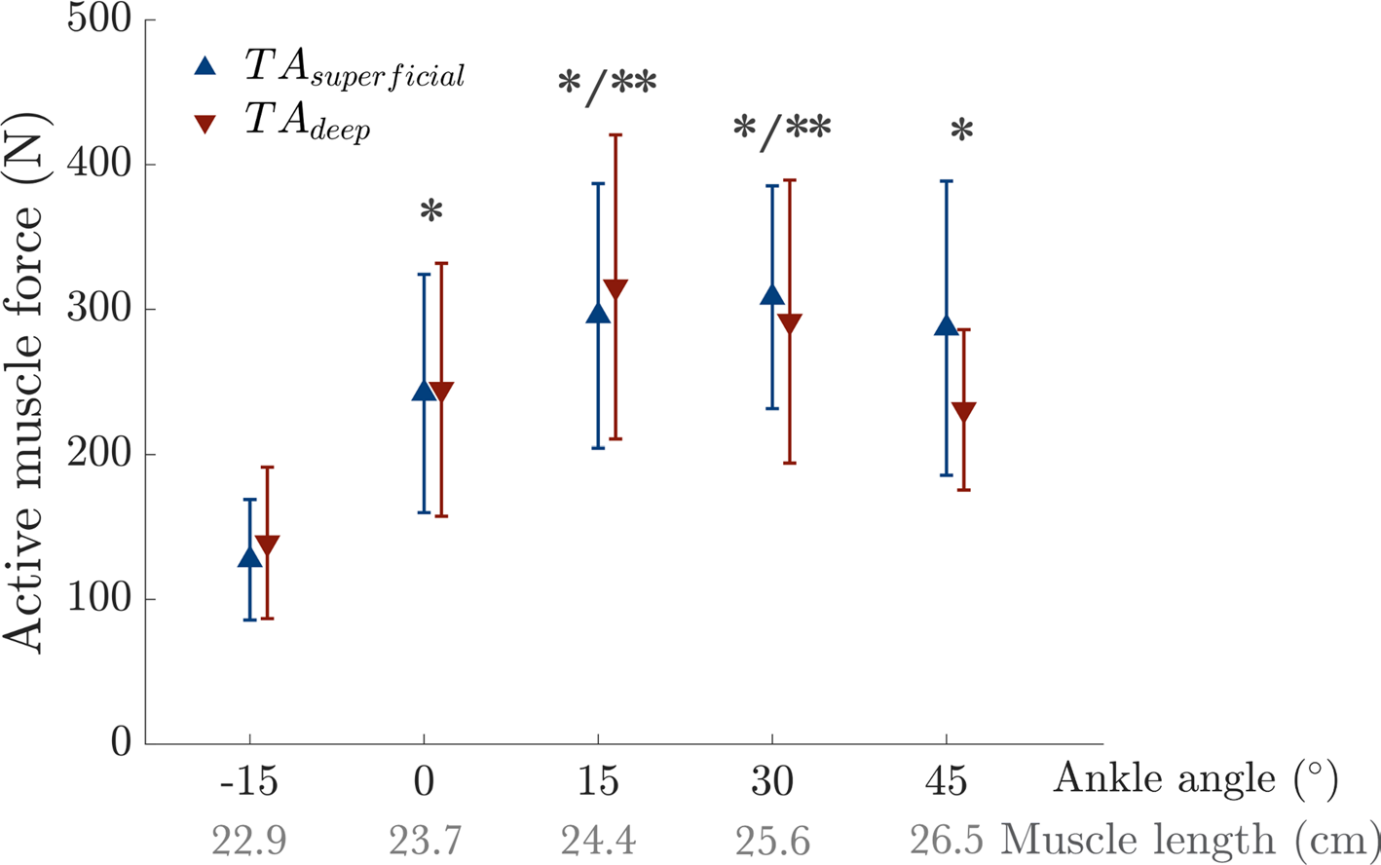
Active force-ankle angle (force–length) relationships for the TA muscle superficial and deep compartments at maximum voluntary contraction. * and ** indicate significant differences from the values measured at the dorsiflexion position at − 15° and 0° ankle angles, respectively.

Active force-ankle angle (force–length) relationships (Fig. 5) were significantly affected only by the ankle angle (F(4, 52) = 37.51, *p* < 0.001), but not by compartment (F(1, 13) = 0.41, *p* = 0.534), with a significant interaction effect was observed (F(4, 52) = 3.58, *p* = 0.012). Post hoc analysis indicated significant differences between − 15° and all other ankle angles (*p* < 0.001 for all) and between 0° and 15° (*p* < 0.001) and 30˚ (*p* = 0.002).

**Fig. 6:**
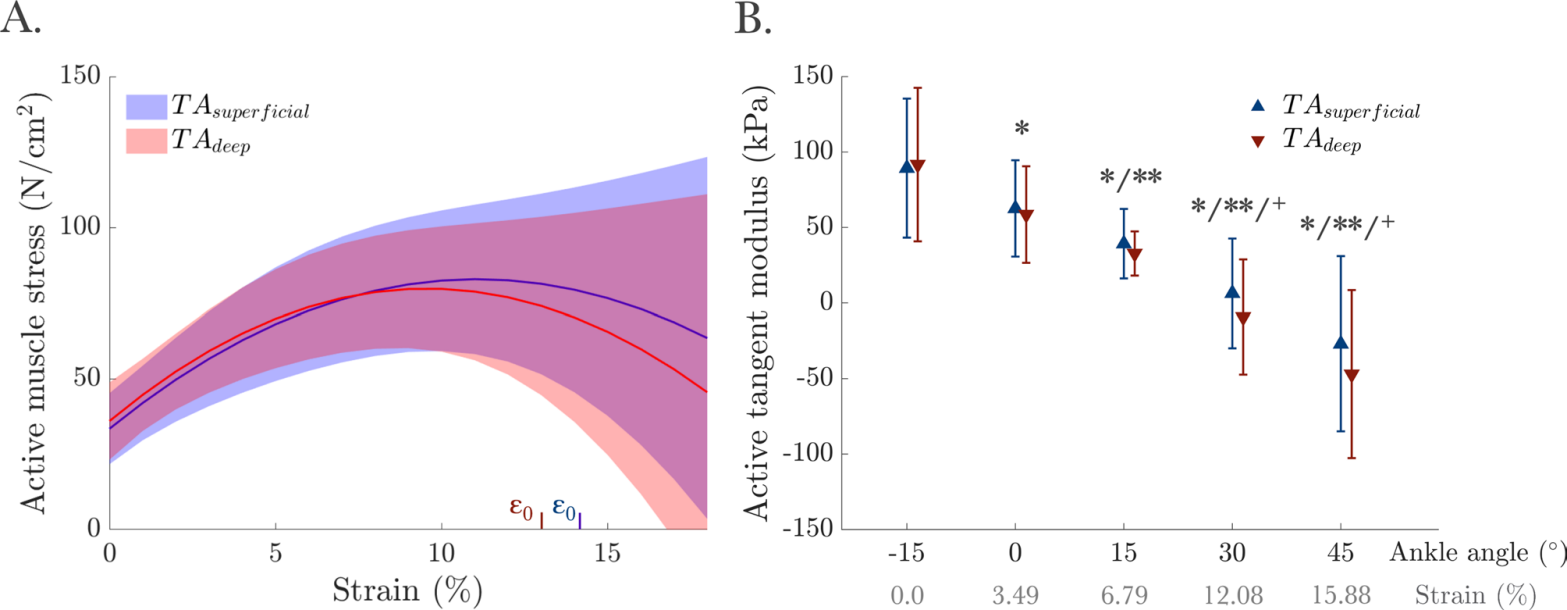
(**A**) Stress–strain and (**B**) Tangent modulus-ankle angle (strain) relationships for the TA muscle superficial and deep compartments at maximum voluntary contraction (MVC). In (**A**), solid lines represent the average fit from all participants, while the shaded area represents the standard deviation. ε_0_ marks the average strain at the maximum active stress from each fit (i.e., optimal strain). In (**B**), *, **, and + indicate significant differences from the values measured at − 15°, 0°, and 15° ankle angles, respectively.

The stress–strain relationships (Fig. 6A) revealed optimum strains of ε_0_ = 14.23 ± 5.37% and 13.12 ± 5.33% for the superficial and deep compartments of the TA, respectively. The stress–strain fit functions yielded R^2^ values of 0.84 ± 0.14 for *TA*_*superficial*_ and 0.80 ± 0.29 for *TA*_*deep*_. The maximum stress at the optimum strain was not different between the *TA*_*superficial*_ (96.50 ± 30.41 N/cm^2^) and *TA*_*deep*_ (87.29 ± 18.44 N/cm^2^) (F(1, 13) = 2.21, *p* = 0.161).

The active tangent modulus was extracted as the first derivative of the active stress–strain function at the respective ankle angles (Fig. 6B). Two-way ANOVA (factors: ankle angle and compartment) demonstrated a significant effect of ankle angle (F(4, 52) = 23.27, *p* < 0.001), with no significant differences between *TA*_*superficial*_ and *TA*_*deep*_ (F(1, 13) = 3.59, *p* = 0.080), and no interaction (F(4, 52) = 2.00, *p* = 0.108). The tangent modulus decreased towards PF. Post hoc tests revealed significant differences between − 15° and 0°/15°/30°/45° (*p* = 0.039, *p* = 0.004, *p* = 0.002, and *p* = 0.003, respectively), and between 0° and 15°/30°/45° (*p* = 0.008, *p* = 0.001, and 0.002, respectively) as well as between 15° and 30° (*p* = 0.007) and 45° (*p* = 0.004). On average, the active tangent modulus decreased by 127.54 kPa from − 15° to 45°.

## Discussion

This study utilized SWE, alongside ultrasound imaging, EMG recordings, and ankle joint moment measurements, to investigate the TA muscle’s in vivo mechanics. By analyzing the TA in the passive state and during various isometric contractions across ankle positions, and quantifying the impact of muscle length and contraction intensity on shear modulus, we sought to gain a comprehensive understanding of its role in ankle joint mechanics and the contribution of SWE to this understanding. Our findings confirmed that the passive shear modulus of the TA muscle increased with muscle length, while the total shear modulus reflected changes in contraction intensities. These findings emphasize the potential of SWE in characterizing in vivo muscle mechanics. With additional validation, SWE-based predictions could advance the study of muscle behavior, serving as a valuable tool for diagnosing and monitoring muscle function and performance.

### Passive muscle characteristics

At rest, only the muscle, not the tendon, lengthened within the TA muscle–tendon unit as the ankle angle extended from DF to PF (Table 1), indicating that each ankle angle tested corresponds to various TA muscle lengths. This lengthening was accompanied by increases in both the passive ankle joint moment (Fig. 1A) and the passive shear modulus of the TA (Fig. 1B), indicating that the TA became stiffer as it was stretched.

The shear modulus of a muscle reflects its elasticity and can be calculated as the slope of the shear stress–strain curve. A higher shear modulus indicates greater stiffness and resistance to deformation under applied shear stress. Previous studies have consistently reported a rise in shear modulus for triceps surae muscles during passive DF^26,37^ and for the TA muscle with increasing PF^15,27,38^. Our findings, which show increased TA passive shear modulus as the muscle lengthened, align with these established observations.

Over the past decade, shear modulus has gained attention as an index for estimating individual muscle force e.g.,^39^. Assuming a consistent relationship between passive shear modulus and muscle force^26,39,40^, our study supports the potential of using shear modulus to predict passive forces. Animal studies have shown that passive force increases non-linearly with muscle length, rising exponentially beyond the muscle’s optimum lengths^41,42^. The similarity between the shear modulus-ankle angle relationship and the passive force–length characteristics reinforces the utility of SWE for estimating passive forces in vivo. For example, Koo et al.^15^ demonstrated that the human TA’s shear modulus remained constant in DF until the ankle exceeded the slack length (10.6° PF), at which point it increased exponentially. Similarly, in this study, the passive shear modulus rose exponentially beyond the neutral ankle position. In the context of a force–length curve, the length at which passive force begins to increase significantly typically coincides with the muscle’s optimum active force production. Maganaris et al.^43^ estimated that the TA operates in vivo on the ascending limb and plateau region of its length–force curve, with optimal force around 30°. Our findings (Fig. 5) corroborate this, indicating that maximum TA active force occurs at 15° to 30°. Given that the shear modulus of the TA during MVC remains unchanged across different ankle positions (Fig. 3A), the TA may be operating near its optimum length, which may explain the lack of change in its stiffness. Thus, SWE holds promise for directly reflecting pre-stress and force in individual muscles.

Passive muscle characteristics have been assessed through direct force–length measurements in animals at fiber^44,45^ and whole-muscle^46,47^ levels. However, such invasive measurements are impractical in humans, where passive joint moment–angle relationships are used instead^48^. While advancements have improved muscle force estimation accuracy, limitations persist, particularly in accounting for both dorsi- and plantar flexor muscles^49,50^. Assumptions regarding agonist–antagonist muscle contributions, varying resting lengths and associated pre-stress of muscles acting at the same joint, and force transmission through intermuscular mechanical interactions complicate these predictions. For example, in rats, triceps surae force decreased by 16% when antagonists were lengthened^51^. Similar interactions between antagonist muscles, TA and GL, have been observed in humans^27^. A study utilizing SWE^27^ reported that the TA shear modulus was lower when the knee was flexed to 90°, compared to full knee extension, despite the TA not spanning the knee. This finding highlights intermuscular mechanical interactions that current techniques cannot account for when calculating passive muscle force from the joint moment. These complexities limit the accuracy of passive force–length relationships and emphasize the need for improved non-invasive methods to assess muscle forces in humans. If validated with direct in vivo force measurements^6,7,9^, SWE could provide a reliable, non-invasive method for estimating passive muscle forces, enhancing the accuracy of biomechanical and musculoskeletal models.

### Active muscle characteristics

During MVC, the active ankle DF moment was minimal at the maximum DF position (− 15°) and increased towards PF (Fig. 2), reaching its highest measured value at a PF position (15°). These findings align with previous studies showing a bell-shaped ankle joint moment with a broad peak between neutral and PF positions^19,20,52–54^. Sasaki et al.^19^ observed similar trends and also showed increasing fascicle length and decreasing pennation angle towards the PF direction. Although they tested a narrower ankle ROM (15° DF to 25° PF), they found a linear increase in total shear modulus with ankle angle. They attributed these findings to the nonlinear elastic properties of connective tissue in series with muscle fibers, which also affect the passive shear modulus. In contrast, our study found no variation in the total shear modulus of the TA with ankle angle during MVC (Fig. 3A). However, during submaximal ramp contractions (Fig. 4A), it was significantly affected by ankle angle, possibly reflecting increased passive stiffness. Alternatively, differences in contraction characteristics, i.e., brief maximal versus slow submaximal contractions, may have also influenced muscle mechanics (as discussed below).

Changes in TA EMG amplitude with ankle angle during MVC (Fig. 3B) are noteworthy. Force production during voluntary contractions varies with muscle length and is influenced by central (e.g., neural activation, motor unit discharge, and recruitment rate) and peripheral (e.g., actin-myosin overlap and Ca^2+^ sensitivity) factors^55,56^. Studies on the motor unit activity at different muscle lengths show conflicting results, with some reporting higher^57^, constant^58^, or lower^59^ neural input at shorter lengths. These variations in EMG amplitude are partially due to peripheral mechanisms and motoneuron discharge and recruitment properties. Mixed findings have also been reported for the TA. Pasquet et al.^60^ found that higher discharge rates and motor-unit recruitment are required at shorter muscle lengths, which aligns with our observation of decreased TA EMG amplitude with increasing muscle length. In addition to muscle length, changes in pennation angle should also be considered when interpreting variations in EMG amplitude. A smaller pennation angle, aligning fibers more parallel to the force direction axis, facilitates more efficient force transmission. Previous studies show a relationship between pennation angle and EMG amplitude, though its nature varies with contraction intensity and methodological factors. For instance, Hodges et al.^61^ reported a nonlinear increase in pennation angle with EMG, particularly at low contraction intensities, while Manal et al.^62^ observed a more linear relationship across contraction levels (from rest to MVC), with a strong correlation in TA (R2 = 0.76). In our study, both pennation angle (Supplement 1) and EMG amplitude decreased with increasing ankle angle, suggesting that larger pennation angles require greater muscle activation to achieve the same level of force output. However, it should be noted that higher EMG amplitudes at more dorsiflexed ankle angles do not necessarily indicate increased neural drive at shorter muscle lengths due to the inherent limitations of low-density surface EMG^63^. Specifically, for muscles like the TA, which undergo complex activation patterns and have a larger volume beneath the electrode surface at more dorsiflexed positions, these limitations are particularly relevant. The low-density nature of surface EMG can lead to issues such as amplitude cancellation, which may mask or distort the true neural drive^64^. These EMG-related findings and potential technical limitations underscore the combined role of muscle architecture and neural control in activation strategies. Future studies utilizing high-density EMG could provide precise tracking of individual motor units across a wide range of ankle angles by improving spatial resolution, reducing amplitude cancellation, and offering a more accurate and detailed measure of neural drive and motor unit activity. Incorporating simultaneous measurements of fascicle behavior and motor unit activity could further elucidate this interaction.

The active ankle joint moment plateaued at around 15° PF, corresponding to longer muscle lengths. Interestingly, the shear modulus of the TA remained constant (Fig. 3A) despite a simultaneous decrease in EMG amplitude (Fig. 3B). This may reflect not only the material properties of the TA muscle itself but also a redistribution of force generation among dorsiflexor muscles as joint position changes. This suggests the involvement of complex force-sharing strategies that adjust based on the joint angle. Prior studies have reported angle- and task-dependent variations in shear modulus among triceps surae muscles^65^. Future work employing simultaneous SWE measurements across dorsiflexor and plantar flexor groups could further reveal such mechanisms in vivo. Accordingly, the combined use of SWE and EMG can enhance our understanding of muscle mechanics by simultaneously capturing mechanical and neural aspects of muscle function. While SWE holds promise as a proxy for muscle stiffness, particularly if its relationship with force output can be further validated, EMG remains essential for tracking activation patterns, especially when joint positioning or architectural changes influence recruitment. Together, these tools can be informative in evaluating muscle adaptations due to exercise, neuromuscular impairments, or musculoskeletal pathology.

During submaximal ramp contractions, the TA total shear modulus effectively distinguished differences in muscle stiffness across all contraction intensity comparisons. These differences were ankle angle-dependent, with significant distinctions observed between 25% vs. 50% MVC and 25% vs. 75% MVC at all ankle angles. However, differences between 50% vs. 75% MVC were only detected at − 15°. Meanwhile, TA EMG amplitude (Fig. 4B) showed significant differences across all contraction levels at all ankle angles, suggesting a stronger link between TA electrical activity and ankle dorsiflexion. These findings may explain the non-linear differences in the shear modulus between 50 and 75% MVC. Intrinsic muscle properties, such as fiber type and sarcomere length, along with anatomical factors (e.g., muscle geometry and positioning), likely contribute to this behavior.

The lower shear modulus observed during MVC compared to some submaximal ramp contractions aligns with the findings of Zimmer et al.^21^, which showed that shear modulus from the biceps brachii was higher during isometric ramp contractions than in MVC trials. This suggests that, beyond joint position and contraction intensity, contraction rate (i.e., short maximal vs. slow submaximal contractions) also influences muscle mechanics. The MVC trials involved a fast ballistic contraction, whereas the submaximal contractions were performed using a controlled ramp-up, likely impacting muscle–tendon dynamics and neuromuscular activation. Rapid and ramp isometric MVCs engage distinct motor control strategies^66^, with rapid contractions requiring abrupt muscle activation, while ramp contractions allow for more gradual and uniform force development. This difference is reflected in higher EMG amplitudes during isometric ramp MVCs compared to ballistic contractions^67,68^. Furthermore, the faster contraction during MVC may have resulted in less uniform muscle activation, altered muscle–tendon interaction, and reduced passive tension contribution, all of which could contribute to the lower active shear modulus at peak force. These findings underscore the significant influence of contraction rate on SWE measurements and highlight the need for further research into how contraction dynamics affect muscle shear modulus.

The active force–length characteristics of the TA (Fig. 5) were derived from ankle DF moments measured during isometric MVCs, incorporating key architectural parameters (cross-sectional area, fascicle length, and pennation angle). Our finding of optimal force production occurring between 15° and 30° falls between previous reports of peak force generation at 10°–15°^20,69^ and at approximately 30°^43^. The tangent modulus-ankle joint characteristics (Fig. 6B), calculated from stress–strain curves (Fig. 6A), were used as a measure of TA stiffness. The stress–strain relationships indicate that the TA operates at lengths ranging from shorter-than-optimal to optimal, with the optimal strain (i.e., strain at maximum stress) near the maximum strain measured. The tangent modulus decreased from 90.51 kPa at − 15° to − 37.03 kPa at 45°, reflecting a reduced slope of the stress–strain curve as maximum stress was approached. Negative values at PF positions align with the descending limb of the stress–strain curve, where high strain (e.g., 15.88% elongation from DF to PF) may amplify these effects. The negative stiffness does not occur physiologically: During rapid length changes, muscles operate in a transient state, in which actin-bound myosin cross-bridges stretch before releasing, generating a transient force known as short-range stiffness. This transient force exceeds the steady-state force predicted by the stress–strain curve, maintaining positive stiffness even on the descending limb^70^. Thus, the observed negative stiffness reflects the steady-state behavior of the TA muscle under isometric conditions and highlights the biomechanical limitations of the TA muscle in PF positions, but does not imply functional instability during natural movements, where transient dynamics dominate.

Unlike this varying tangent modulus (Fig. 6B), the shear modulus did not change with strain (122.96 ± 9.87 kPa across all ankle angles, Fig. 3A). This contrast highlights differences between these techniques in assessing muscle stiffness. Tangent modulus depends on the force–length relationship, muscle architecture assumptions, and force-sharing across joint positions, whereas SWE measures shear modulus via wave propagation theory, assuming linear elasticity. These differences may stem from methodological assumptions or reflect actual mechanical disparities. Further validation of both approaches and their assumptions is necessary to clarify TA muscle mechanics.

### Limitations of shear wave elastography

The limitations of the SWE technique must be considered when interpreting these findings. The percentage of pixels reaching the system’s maximum measurable SWV (14.1 m/s) and corresponding shear modulus (200 kPa) likely varied across conditions. This variation could have led to an underestimation of the shear modulus, particularly in trials with higher contraction intensities and at more plantarflexed joint angles (see Supplement 3). During MVC, average total shear modulus values remained well below this limit, for example, 122.96 ± 9.87 kPa on average, with a maximum of 139.76 ± 24.91 kPa at 45°. Notably, across ankle angles, the proportion of such pixels was lowest at 0° (15.96 ± 11.62%) and increased progressively toward 45° (28.57 ± 15.60%), where the highest occurrence was observed. This suggests that joint position can influence the extent to which values approach the measurement ceiling, which may, in turn, affect the interpretation of muscle shear modulus at more plantarflexed positions. These observations partially contrast with findings from Sasaki et al.^19^, who reported a linear increase in total shear modulus from 15° DF to 25° PF. We observed no differences in the percentage of pixels reaching the maximum value within the ROI at this ankle angle range (maximally 19.95 ± 12.64% at − 15°). While saturation levels were comparable, the effect of these saturation artifacts on the detection of angle-dependent differences in shear modulus remains uncertain. That said, our findings are more consistent with those of Zimmer et al.^21^, who studied the biceps brachii and found no significant differences in shear modulus across joint angles during MVC. In their study, average shear modulus values (~ 60 kPa, well below the measurement ceiling) suggested that the lack of angle-related differences was physiological rather than technical.

Similarly, during submaximal ramp contractions, the percentage of pixels reaching 200 kPa increased with both plantarflexed joint angle and contraction intensity (Supplement 3). This trend was most apparent at higher intensities (50% and 75% MVC), particularly at 30° and 45°. Despite this, differences in total shear modulus were still observed between certain ankle angles at both 50% and 75% MVC. Similarly, although the percentage of pixels reaching the maximum shear modulus value increased with contraction intensity, significant differences were still evident between all intensity levels, including between 50% vs. 75% MVC. Notably, Zimmer et al.^21^ also found no significant difference in biceps brachii shear modulus between 50 and 75% MVC, despite values remaining below the system limit. They attributed this to physiological factors, suggesting that the biceps brachii reaches near-maximal activation before peak moment generation, with further increases supported by synergists rather than additional activation. A comparable explanation could be considered for the TA: as intensity exceeds 50% MVC, it is possible that additional joint moment is supported by synergists, potentially limiting further increases in TA force (consistent with the observed TA force patterns, Fig. 5). Furthermore, at higher contraction intensities, participants may generate small moments at adjacent joints, such as hip flexion in the supine position, which could contribute to the measured net ankle joint torque. Although participants were instructed to restrict movement to the ankle joint, mechanical coupling between joints can still lead to unintended joint moments during multi-joint tasks^71–74^. As rectus femoris activity was not recorded, we cannot confirm this effect, but such unintended contributions may partially explain discrepancies between net torque and TA-specific measures. Redistribution of force (either among synergists crossing the ankle or due to activation of muscles acting at adjacent joints) may, in part, explain the lack of consistent differences between 50 and 75% MVC across joint angles. However, this interpretation should be approached cautiously, as the relatively high proportion of saturated pixels at 75% MVC should have contributed to an underestimation of the total shear modulus, potentially influencing the absence of differences at certain joint angles (except at − 15°). Collectively, these findings suggest that SWE-based estimates of TA muscle mechanical properties are more robust at lower contraction intensities, where signal saturation is less prevalent. At higher intensities, however, the reliability of SWE measurements may be compromised by increased saturation, complicating the interpretation of absolute shear modulus values. While our saturation analysis (Supplement 3) provides important context, we acknowledge that pixel saturation results from acquisition-related hardware limitations (e.g., system measurement ceiling) that cannot be resolved retrospectively. Given these constraints, future studies should interpret high-intensity SWE data with caution and consider the use of improved ultrasound systems or complementary assessment methods. Specifically, to more definitively assess the contributions of individual muscles and potential force-sharing among synergists, simultaneous elastographic assessment of multiple dorsiflexor muscles may help clarify the extent of force redistribution among synergists across a range of joint angles and contraction intensities.

Furthermore, SWE provides localized estimates of tissue mechanics and is sensitive to factors such as pennation angle and regional heterogeneity, both of which may vary with joint position and contraction level. For instance, a recent study on the gastrocnemius medialis suggested that increased pennation at higher contractions can alter shear wave propagation by enhancing the deflection of faster shear waves^29^. However, no significant effects were observed when the ultrasound probe was aligned with muscle fibers^29^. Although we aimed to maintain consistent transducer placement over the same muscle region, minor shifts likely occurred due to muscle geometry changes. While we did not specifically assess ROI depth effects, existing literature offers mixed findings; some report lower SWV at greater depths^75^, whereas others find no such effect^76^. If deeper placement did occur at higher intensities, the true shear modulus may be underestimated, potentially amplifying the influence of the pennation angle on measurement outcomes.

Additionally, minor joint rotations during isometric contractions cannot be entirely ruled out. While a fixed experimental setup was used to minimize deviations from the prescribed ankle positions, Raiteri et al.^77^ reported slight deviations in ankle angle during DF contraction. In vivo isometric contractions do not imply fixed fascicle length due to the elastic properties of tendons and aponeuroses^78^. While we refer to the contractions as isometric at the joint level, fascicle shortening likely occurred. Muscle architectural changes such as this fascicle shortening, occurring even when muscle–tendon unit length remains relatively constant^20,38^, influence muscle mechanics in ways not fully captured by gross muscle–tendon length or work. Our methodology, however, focuses on presenting muscle and joint-level data concerning ankle joint position rather than fascicle length. Consequently, the shear modulus reported under activated conditions here reflects the total shear modulus, combining both passive and active components. Future studies estimating active shear modulus from the total and validating these estimates against stiffness measured using complementary techniques (ideally with in vivo data) could provide deeper insights into pure contractile behavior. By relating shear modulus to ankle joint position, and ultimately, in future studies, to muscle architectural parameters, SWE could provide valuable insights into these mechanisms, enhancing our understanding of muscle function during movement.

### Limitations of in vivo muscle force estimation

Estimating muscle force from the joint moment requires accounting for the tendon moment arm^20,43^ and the ratio of a muscle’s PCSA to the total PCSA of the muscle group^20^. While animal studies showed a linear correlation between PCSA and maximum tetanic forces^79^, the accuracy of this approach in distinguishing individual muscle contributions in vivo remains debated. Several assumptions underlie this method. First, it assumes a uniform specific force across all muscles at a given joint position. Second, the correlation between PCSA and maximum tetanic force is typically based on measurements at the muscle’s optimum length^79^. In vivo, however, muscles often operate across different segments of their active force–length curve depending on the joint position. Although PCSA may be a reliable predictor of maximum force at a muscle’s optimum length, it does not account for the length-dependent characteristics of force production. For instance, in a study examining the correlation between PCSAs of the TA and EDL and their forces, a significant relationship was observed only for the TA (*p* = 0.80), not for the EDL (*p* = 0.41), and the correlation for the DF muscle group was moderate (*p* = 0.70)^80^. Moreover, the correlation between the PCSA and force varied depending on joint positions. In particular, for TA, the correlation increased with greater ankle angles, rising from *p* = 0.29 at 0° to *p* = 0.80 at 30°. This finding underscores the limitations of PCSA as a single predictor of muscle force across all joint positions. Furthermore, PCSA-based estimates do not consider passive force generation or the pre-stress in muscles. This shortcoming indicates the difficulty of calculating passive force–length characteristics of TA from the measured passive ankle joint moment. In contrast, SWE has the potential to provide more nuanced insights into muscle mechanics once validated.

In this study, we calculated the PCSA for each individual separately, accounting for both compartments of the TA and using each subject’s specific fascicle length and pennation angle data. Muscle volume was estimated from subject-specific ACSA and muscle length measurements obtained through ultrasound at each tested ankle angle. Our calculations used a shape factor derived from literature values for the entire TA, applied uniformly to both compartments of the TA, specific to each subject. This approach, though necessary due to the lack of individual-specific volume information, introduces a limitation. Applying more imaging techniques, such as 3D ultrasound^81^, could improve accuracy by providing more precise structural information on both the muscle and its compartments. Lastly, the tendon moment arms used in our calculations were derived from literature, as directly measuring them during MVC would be time-consuming and infeasible. However, these literature values are based exclusively on male subjects^1,82^ and may vary due to differences in measurement methods or individual subject variations. For example, moment arm lengths were not adjusted according to the anthropometric measurements in this study, as body size and joint dimensions do not reliably predict moment arm lengths^83^.

During MVC, fascicles from both TA compartments shorten similarly, producing comparable force and work at the same ankle angle. Although the deep compartment had larger pennation angles at certain positions, these differences did not significantly affect the resultant compartmental muscle forces. Notably, the ACSA of the midsection of the TA decreased by 18% as the muscle lengthened from − 15° to 45°. This change may influence shear modulus measurements by altering intramuscular load distribution and force transmission. However, since no differences in force and stress were observed between compartments, SWE measurements predominantly taken from the superficial compartment can be considered representative of the entire TA muscle.

## Conclusion

This study evaluated the in vivo mechanical characteristics of the TA muscle and its role in ankle joint mechanics. SWE findings indicated that the TA’s passive shear modulus increases with muscle length, suggesting SWE’s potential as a tool for measuring pre-stress and estimating passive muscle force. During activation, SWE captured varying contraction levels. However, interpreting these results remains challenging due to the complex force-sharing dynamics among muscles at different joint positions and activation levels. The joint moment-driven in vivo force–length properties revealed maximal TA force at 15°–30°, suggesting that TA operates on the ascending limb and within a constant region of its force–length curve during isometric MVCs. Comparing ankle joint moment-derived tangent modulus characteristics with shear modulus obtained using SWE presented discrepancies. These highlight the need for validation of both estimation approaches.

## Methods

### Participants

Fourteen healthy young adults (7 females and 7 males; age: 26.43 ± 3.67 years) provided written informed consent before participating in the study. None were taking medications that could affect muscle strength. Participants were asked to refrain from strenuous activities 24 h before the study to eliminate potential exercise effects.

### Anthropometrics and muscular architecture

Tibia length and calf circumference were measured using a tape measure with participants standing, while their neutral ankle angle and passive and active ankle ROM were assessed in a supine position using a goniometer. Muscle–tendon architecture was evaluated using real-time two-dimensional B-mode ultrasound (AixPlorer Mach 30, MSK preset, Supersonic Imagine, Aix-en-Provence, France) with a linear transducer (2–10 MHz; 38 mm footprint; L10-2; Supersonic Imagine, Aix-en-Provence, France). Muscle length, tendon length, and ACSA of the TA muscle were measured at ankle angles of − 15°, 0°, 15°, 30°, and 45° with participants in a supine position. A custom-built footplate was used to maintain the desired ankle angle, which was measured using a goniometer. Muscle and tendon lengths were determined using anatomical landmarks: the TA’s origin on the lateral surface of the tibia and its insertion on the medial cuneiform bone. Markers placed on the skin guided the measurement process. Muscle length was measured from the origin to the myotendinous junction, and tendon length from the myotendinous junction to the bone insertion. The ACSA was measured from the widest part of the TA muscle.

### Experimental measurements

**Fig. 7:**
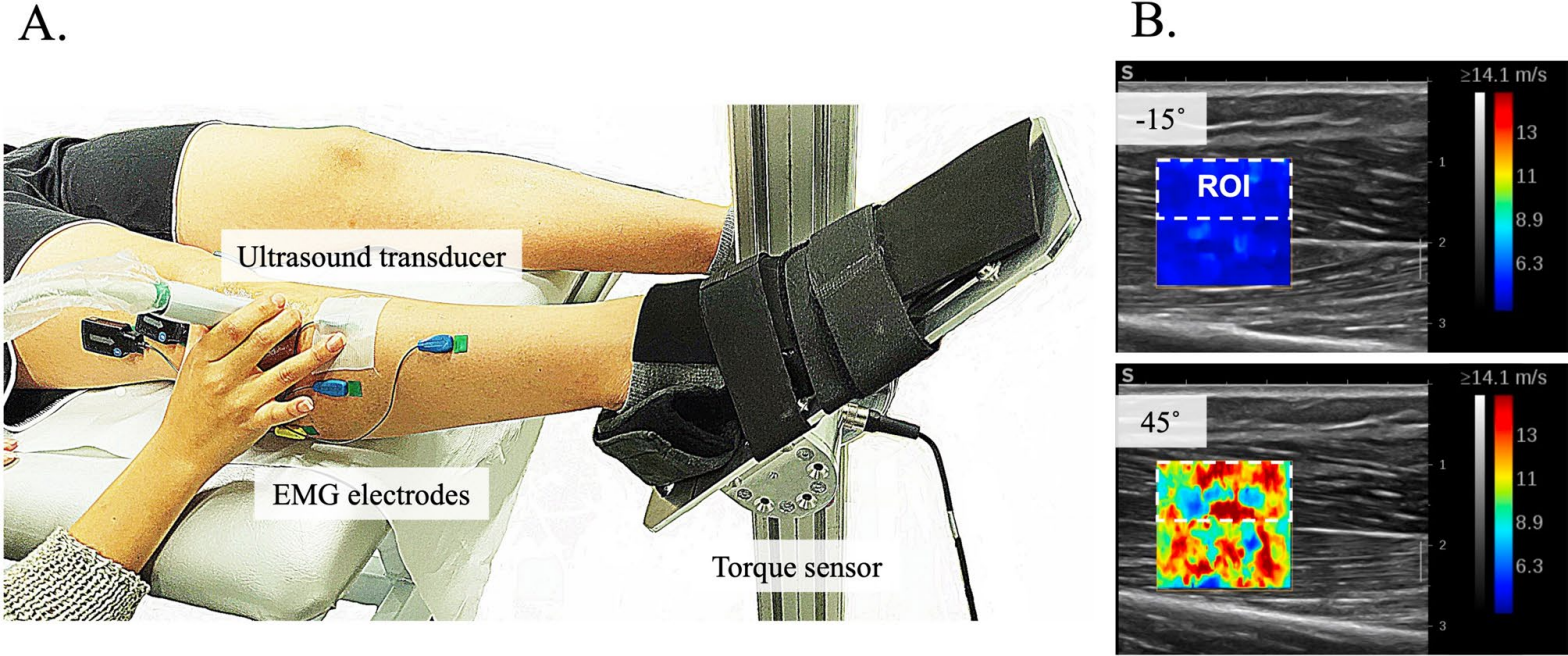
(**A**) Experimental setup with a participant in a supine position. The ankle joint is fixed at a 45° plantar flexion (PF) ankle angle using a custom-built footplate. Measurements are performed at ankle angles of − 15°, 0°, 15°, 30°, and 45°. The center of rotation of the ankle joint is aligned with the torque cell attached to the apparatus. Surface electromyography (EMG) electrodes placed on the tibialis anterior (TA), extensor digitorum longus (EDL), peroneus longus (PERL), and gastrocnemius lateralis (GL) are shown, while the ones placed on the gastrocnemius medialis (GM) and soleus (SOL) muscle at the posterior are not visible on the picture. The ultrasound transducer is placed on top of the TA muscle belly and aligned longitudinally with the TA muscle. (**B**) Example ultrasound frames, including shear wave velocity (SWV) color overlays and selected region of interest (ROI) during 25% MVC isometric ramp contractions for dorsiflexion (at − 15°) and PF (at 45°) positions.

Simultaneous measurements of ankle joint moment, EMG signals, and ultrasound videos (B-Mode and SWV maps) were obtained from the right leg of all participants, which was identified as their self-reported dominant leg based on the preferred kicking leg. Participants were positioned supine on an examination bed with their knees fully extended and feet on a plate (Fig. 7A).

*Joint moment* A custom-made apparatus equipped with a static torque sensor (DF30, 200 Nm, Lorenz Messtechnik GmbH, Aldorf, Germany) was used to measure the isometric ankle joint moment. The apparatus allowed fixation of the ankle at specific angles, verified with a digital inclinometer. Participants were positioned with their feet secured to the plate, ensuring that the center of rotation of the ankle joint was aligned with the center of the torque sensor. The bed height was adjusted to keep the tibia parallel to the floor at each ankle angle, and the footplate was fixed at the desired ankle angles. Velcro straps were used to restrict ankle movement.

*Surface EMG* During the experiment, the electrical activity of the TA muscle, extensor digitorum longus (EDL) in the same compartment, peroneus longus (PERL) in the lateral compartment, and gastrocnemius medialis (GM), gastrocnemius lateralis (GL), and soleus (SOL) in the posterior compartment were monitored. The surface EMG signals were recorded using dry-surface electrodes of the Trigno Wireless Biofeedback System (Delsys Europe, Greater Manchester, UK). All electrodes had a 10 mm inter-electrode spacing and a dual on-board stabilizing reference. The Trigno Duo Sensor was used for GM and GL, while the electrical activity of all other muscles was measured using Trigno Mini Sensors. All electrodes were placed longitudinally along the muscle following the SENIAM recommendations^84^. For the EDL muscle, not covered by SENIAM, electrode placement was guided by anatomical landmarks, with the muscle identified through palpation along the fibular shaft, slightly lateral to the TA, and medial to the peroneal muscles. Due to the simultaneous SWE measurement, the electrode for the TA was placed slightly distal to the central position over the muscle belly.

*SWE* SWV maps were obtained using an ultrasonic scanner equipped with SWE (AixPlorer Mach 30, MSK preset, Supersonic Imagine, Aix-en-Provence, France) and a linear transducer (2–10 MHz; 38 mm footprint; L10-2; Supersonic Imagine, Aix-en-Provence, France). The transducer was positioned longitudinally near the thickest part of the TA muscle, proximal to the EMG electrode. The SWV maps were displayed within a rectangular region of interest (ROI) covering the muscle fascicles (Fig. 7B). The shear modulus (G, in N/m^2^ = Pa) was calculated from the SWV ($v_{s}$), assuming the muscle behaves as a transverse isotropic, linear elastic material in an unstressed state:

1 $$G = \rho \cdot v_{s}^{2}$$

where ρ represents the mass density (with an estimated value of *ρ*_*muscle*_ ≈ 1000 kg/m^3^). A detailed derivation of Eq. (1) can be found in previous literature (e.g.,^85^).

The SWV maps were recorded at 1.5–1.9 Hz, depending on the size and position of the ROI. The maximum SWV (or corresponding shear modulus) provided by the system was 14.1 m/s (or 200 kPa).

The EMG and torque signals were recorded using a data acquisition system (cDAQ-9174, National Instruments, Austin, TX, USA) with a sampling rate of 2 kHz. Synchronization of ultrasound recordings with the data acquisition system was achieved using an Arduino board (Arduino S.r.l., Monza, Italy). Custom MATLAB (The MathWorks, Inc., Natick, MA, USA) scripts were used for data acquisition, visualization, and storage.

### Experimental protocol

The experiments started with a warm-up protocol consisting of isometric ankle DF maximum voluntary contractions (MVCs) and isometric submaximal ramp contractions (from rest to 25%, 50%, and 75% of MVC moment) at two ankle angles (− 15° and 45°). Later, five ankle angles from a DF position at − 15° to a maximal PF position at 45° were examined at 15° intervals. For all participants, the order of ankle angles was consistent. At each ankle angle, the following steps were performed:

*Passive state* (i) Two 5-s recordings were obtained while participants were instructed to fully relax their legs.

*Active state* (ii) Participants performed three 5-s isometric ankle DF MVCs, with a 1-min rest between trials.

(iii) Isometric submaximal ramp contractions were conducted, where participants increased their ankle DF moment following a trapezoidal line: 3 s of rest, a linear ramp-up to 25%, 50%, or 75% MVC (at a slope of 25% per second), and a 7-s constant contraction. Two trials were recorded at each level, always in the order of 25%, 50%, and 75% MVC, with a 30-s rest between trials. Participants followed a line on a screen, with the cursor position representing the real-time ankle moment normalized to the MVC moment. Trial repetitions were performed as needed to ensure accurate tracking and joint fixation.

### Data processing and analysis

Data analysis and statistics were conducted using MATLAB (The MathWorks, Inc., Natick, MA, USA). Trial averages were computed over the last 4 s for resting trials, the last 3 s for MVC trials, and the last 5 s of the constant phase for submaximal ramp contraction trials. For each ankle angle tested, recordings from passive, MVC, and ramp contraction trials were averaged over two, three, and two trial repetitions, respectively, and averages and standard deviations were calculated.

**Fig. 8:**
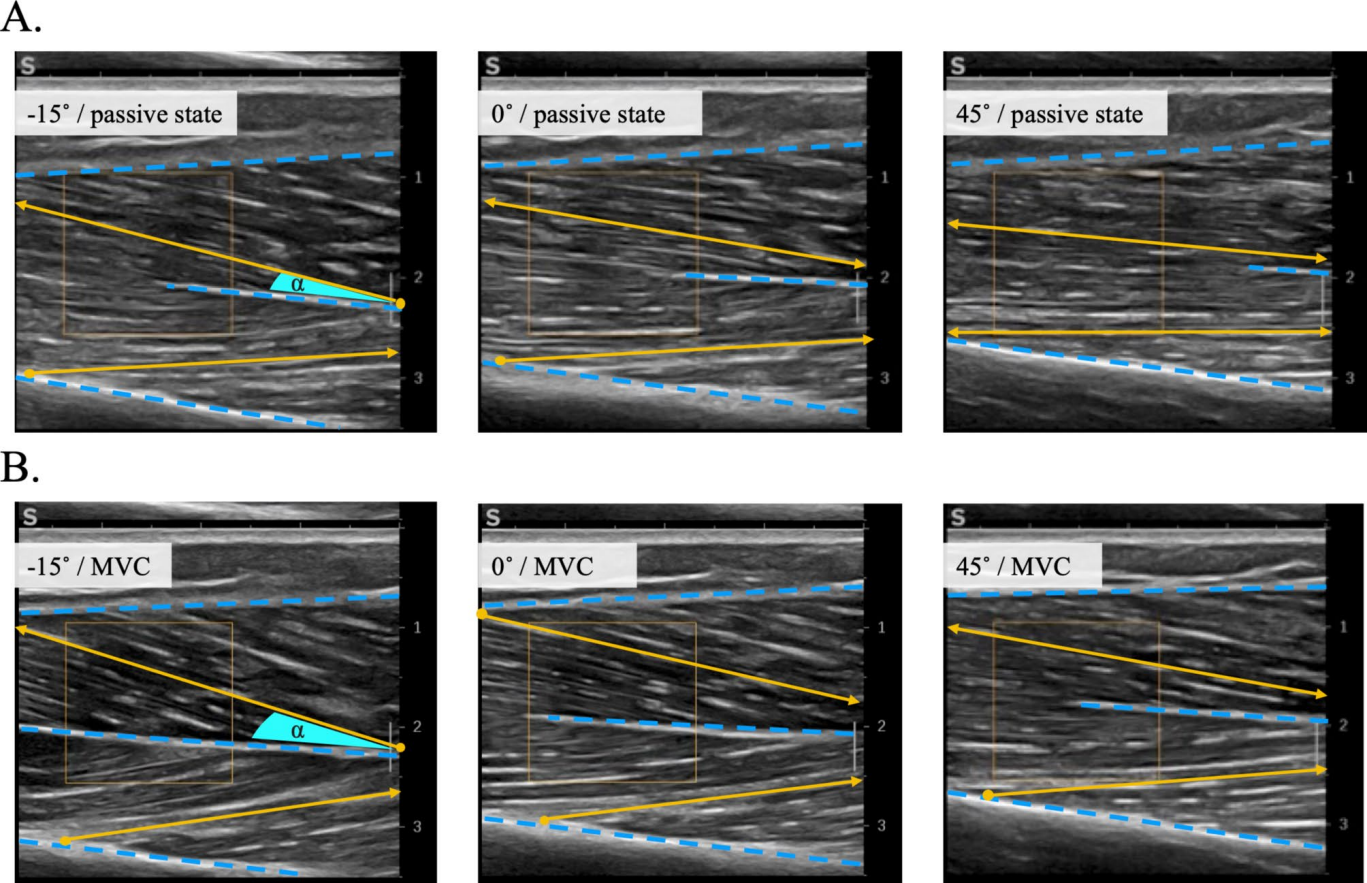
The fascicle length and pennation angle of the TA muscle measured at (**A**) rest and (**B**) maximum voluntary contraction (MVC), shown at three different ankle angles: DF at − 15°, 0°, and PF at 45°. The superficial, central, and deep aponeuroses of the TA are indicated by blue dashed lines. In each view, one fascicle from each compartment is marked by a solid yellow line, with the pennation angle (α) and endpoints (either directly visible or extrapolated, indicated by yellow-filled circles and arrows, respectively) also exemplified.

*Muscular architecture* B-mode ultrasound videos were analyzed using an adapted version of a semi-automatic fascicle tracking algorithm^86^. At the initial frame of each trial, the deep, superficial, and central aponeuroses, along with three fascicles from both deep and superficial compartments, were manually outlined and tracked through the video. From these data, average fascicle lengths and pennation angles (as the angle between the fascicles in the respective compartment and the central aponeurosis) were calculated (Fig. 8).

*Joint moment* The maximum ankle joint moment was calculated from all MVC trials. The active DF moment at each ankle angle was then used to normalize the ankle joint moment during submaximal ramp contractions. Active ankle joint moment was calculated by subtracting the passive joint moment from the total joint moment recorded during contraction at each ankle angle.

*Surface EMG* All EMG recordings were visually inspected for errors (e.g., loose electrodes, operator interference). A fourth-order Butterworth filter (20–250 Hz bandwidth) was applied. As a measure of the EMG amplitude, root mean square values were calculated using 250 ms moving windows. Mean EMG amplitudes during the last 3 s of MVC trials were used to normalize EMG signals from passive and ramp trials. TA and EDL EMG amplitudes were normalized to the dorsiflexion MVC values at each ankle angle, while the plantar flexor muscles were normalized to the EMG amplitudes at plantar flexion MVC at 0° ankle angle. At rest, trials with a normalized EMG amplitude of the TA below 5% were considered passive. Specifically, for each resting trial: (i) shear modulus and muscle architectural measures were excluded if the TA EMG amplitude exceeded 5%, (ii) ankle moment data were excluded if the median EMG amplitude across muscles exceeded 5%, and (iii) individual EMG signals were excluded if a given muscle’s EMG exceeded 5%.

*SWE* Videos were analyzed by decoding the 2D color map into numerical values. For each trial, an ROI was manually selected to include only muscle fascicles while excluding the central aponeurosis and subcutaneous fat (Fig. 7B). Recordings were analyzed frame by frame, and frames with less than 75% valid (i.e., colored) pixels within the ROI were excluded. The percentage of pixels reaching the maximum measurable SWV of 14.1 m/s was determined as a quality measure.

*Deriving active muscle force and stress–strain relationships* Using the ankle joint moments obtained as a function of ankle angle, the TA forces (*F*_*TA*_) at its tendon were estimated by the following equation:

2 $$F_{TA} = \left( {\frac{{M_{ankle} }}{{r_{TA} }}} \right) \cdot 0.5$$

where $M_{ankle}$ represents the active ankle DF moment, and $r_{TA}$ is a joint-angle-specific muscle–tendon moment arm, derived from literature for MVC conditions^1,82^. The factor 0.5 assumes TA contributes 50% of the total DF force based on its PCSA relative to the other dorsiflexors^87,88^.

The forces in the TA’s superficial ($F_{TA\;superficial}$) and deep ($F_{TA\;deep}$) compartments were determined using:

3 $$F_{TA} = F_{TA\;superficial} + F_{TA\;deep}$$

4 $$F_{TA\;superficial} = F_{TA} \left( {\frac{{PCSA_{TA\;superficial} }}{{PCSA_{TA} }}} \right)$$

5 $$F_{TA\;deep} = F_{TA} \left( {\frac{{PCSA_{TA\;deep} }}{{PCSA_{TA} }}} \right)$$

To determine the volume of the TA, the following mathematical model was applied^89,90^:

6 $$V_{TA} = p \cdot ACSA_{TA} \cdot l_{TA}$$

where $p$ calculated to be 0.51 based on published data for TA muscle volume ($V_{TA}$*)*, muscle length ($l_{TA}$), and maximum ACSA ($ACSA_{TA}$)^89,90^. We calculated maximum compartmental ACSA values ($ACSA_{TA\;superficial}$ and $ACSA_{TA\;deep}$) using B-mode ultrasound images taken at the widest middle section of the TA in a 0º ankle position. These calculations accounted for the locations and orientations of the superficial, central, and deep aponeuroses of the TA. For the same ankle position, $l_{TA}$ was also measured on a subject-specific basis. With the formula (6), we calculated compartment-specific muscle volumes ($V_{TA\;superficial}$ and $V_{TA\;deep}$) for each participant.

To calculate compartmental PCSA values for the TA muscle ($PCSA_{TA\;superficial}$ and $PCSA_{TA\;deep}$) on a subject- and ankle-angle-specific basis, we applied compartment-specific fascicle lengths ($l_{fascicle\;superficial}$ and $l_{fascicle\;deep}$) and pennation angles (${\upalpha }_{superficial}$ and ${\upalpha }_{deep}$) obtained for MVC to the following formulas:

7 $$PCSA_{TA\;\sup erficial} = \frac{{V_{TA\;superficial} }}{{l_{fascicle\;superficial} }} \cdot \cos \left( {{\upalpha }_{\sup erficial} } \right)$$

8 $$PCSA_{TA\;deep} = \frac{{V_{TA\;deep} }}{{l_{fascicle\;deep} }} \cdot \cos \left( {{\upalpha }_{deep} } \right)$$

Once the active forces exerted by each TA compartment ($F_{TA\;superficial}$ and $F_{TA\;deep}$) were calculated, using the same compartmental ACSA values ($ACSA_{TA\;superficial}$ and $ACSA_{TA\;deep}$) as described above, we derived the compartment-specific TA muscle stress ($\sigma_{TA\;superficial}$ and $\sigma_{TA\;deep}$):

9 $$\sigma_{TA} = \frac{{F_{TA} }}{{ACSA_{TA} }}$$

Muscle strain was determined based on the TA length change relative to its length at − 15°. Polynomial curve fitting (linear or quadratic, based on a higher coefficient of determination (R^2^)) was used to model stress–strain relationships. From the stress–strain function, 19 data points were extracted from 0 to 18% strain, with 1% intervals. The strain corresponding to the maximum stress within this strain range was designated as the optimal strain (ε_0_). Tangent modulus (i.e., the slope of the stress–strain curve) values were determined by differentiating the stress–strain functions at muscle strains corresponding to measured ankle angles.

## Statistical Analyses

We conducted repeated measures analysis of variance (ANOVA) to evaluate the effects of ankle angle, muscle compartment, or muscle contraction intensity on the derived measures.

Muscle and tendon lengths as well as the ACSA of the TA were analyzed using one-way repeated ANOVA, with the factor being ankle angle (− 15°, 0°, 15°, 30°, and 45°). Fascicle length and pennation angle were compared across two compartments of the TA (superficial vs. deep) using two-way repeated ANOVA, with factors being ankle angle and condition (passive state vs. MVC).

To specifically test the effect of ankle angle during passive and MVC trials, one-way repeated ANOVA was applied. For ramp contractions, two-way repeated ANOVA was applied, with the factors being ankle angle and contraction intensity (25%, 50%, and 75% MVC). Two-way repeated ANOVA was also employed to analyze relationships such as TA muscle force-ankle angle, stress–strain, and tangent modulus-ankle angle, assessing potential compartment effects, in addition to the factor of ankle angle (or, depending on the test, muscle length or strain).

Bonferroni post hoc pairwise comparison tests were conducted to identify significant within-factor differences. F- and *p*-values were reported in the format: F(d1, d2) = F-value, *p* = *p*-value, where d1 and d2 represent the numerator and denominator degrees of freedom, respectively (i.e., F(d1, d2) with d1 = (k-1) and d2 = (N-1) ∙ (k-1) with N representing the number of participants and k representing the number of conditions). Differences were considered significant at *p* < 0.05.

## Supplementary Information

Supplementary Information 1.

Supplementary Information 2.

Supplementary Information 3.

## Data Availability

The datasets generated during and/or analyzed during the current study are available from the corresponding author upon reasonable request.

## References

[1] Maganaris, C. N., Baltzopoulos, V. & Sargeant, A. J. Changes in the tibialis anterior tendon moment arm from rest to maximum isometric dorsiflexion: In vivo observations in man. *Clin. Biomech.* **14**, 661–666 (1999). 10.1016/s0268-0033(99)00018-2 10521650

[2] Kaya Keles, C. S. & Ates, F. How mechanics of individual muscle-tendon units define knee and ankle joint function in health and cerebral palsy—A narrative review. *Front. Bioeng. Biotechnol.* **11**, 1287385 (2023). 38116195 10.3389/fbioe.2023.1287385 PMC10728775

[3] Ates, F., Temelli, Y. & Yucesoy, C. A. Human spastic gracilis muscle isometric forces measured intraoperatively as a function of knee angle show no abnormal muscular mechanics. *Clin. Biomech.* **28**, 48–54 (2013). 10.1016/j.clinbiomech.2012.08.012 23021616

[4] Persad, L. S. et al. Procedures for obtaining muscle physiology parameters during a gracilis free-functioning muscle transfer in adult patients with brachial plexus injury. *Sci. Rep.* **12**, 6095 (2022). 35414165 10.1038/s41598-022-09861-y PMC9005531

[5] Yucesoy, C. A., Ates, F., Akgun, U. & Karahan, M. Measurement of human gracilis muscle isometric forces as a function of knee angle, intraoperatively. *J. Biomech.* **43**, 2665–2671 (2010). 20655048 10.1016/j.jbiomech.2010.06.002

[6] Kaya, C. S., Bilgili, F., Akalan, N. E. & Yucesoy, C. A. Intraoperative testing of passive and active state mechanics of spastic semitendinosus in conditions involving intermuscular mechanical interactions and gait relevant joint positions. *J. Biomech.* **103**, 10955 (2020). 10.1016/j.jbiomech.2020.109755 32204891

[7] Kaya, C. S. et al. Intraoperative experiments combined with gait analyses indicate that active state rather than passive dominates the spastic gracilis muscle’s joint movement limiting effect in cerebral palsy. *Clin. Biomech.* **68**, 151–157 (2019). 10.1016/j.clinbiomech.2019.06.005 31212210

[8] Yucesoy, C. A., Temelli, Y. & Ates, F. Intra-operatively measured spastic semimembranosus forces of children with cerebral palsy. *J. Electromyogr. Kinesiol.* **36**, 49–55 (2017). 28735102 10.1016/j.jelekin.2017.07.003

[9] Brendecke, E., Tsitlakidis, S., Götze, M., Hagmann, S. & Ates, F. Quantifying the effects of achilles tendon lengthening surgery: An intraoperative approach. *Front. Physiol.* **14**, 1143292 (2023). 36950296 10.3389/fphys.2023.1143292 PMC10025307

[10] Kaya, C. S., Temelli, Y., Ates, F. & Yucesoy, C. A. Effects of inter-synergistic mechanical interactions on the mechanical behaviour of activated spastic semitendinosus muscle of patients with cerebral palsy. *J. Mech. Behav. Biomed. Mater.* **77**, 78–84 (2018). 28892760 10.1016/j.jmbbm.2017.08.040

[11] Ates, F., Temelli, Y. & Yucesoy, C. A. Effects of antagonistic and synergistic muscles’ co-activation on mechanics of activated spastic semitendinosus in children with cerebral palsy. *Hum. Mov. Sci.* **57**, 103–110 (2018). 29197788 10.1016/j.humov.2017.11.011

[12] Arnold, E. M., Ward, S. R., Lieber, R. L. & Delp, S. L. A model of the lower limb for analysis of human movement. *Ann. Biomed. Eng.* **38**, 269–279 (2010). 19957039 10.1007/s10439-009-9852-5 PMC2903973

[13] Out, L., Vrijkotte, T. G. M., van Soest, A. J. & Bobbert, M. F. Influence of the parameters of a human triceps surae muscle model on the isometric torque-angle relationship. *J. Biomech. Eng.* **118**, 17–25 (1996). 8833070 10.1115/1.2795940

[14] Erdemir, A., McLean, S., Herzog, W. & van den Bogert, A. J. Model-based estimation of muscle forces exerted during movements. *Clin. Biomech.* **22**, 131–154 (2007). 10.1016/j.clinbiomech.2006.09.005 17070969

[15] Koo, T. K., Guo, J. Y., Cohen, J. H. & Parker, K. J. Quantifying the passive stretching response of human tibialis anterior muscle using shear wave elastography. *Clin. Biomech.* **29**, 33–39 (2014). 10.1016/j.clinbiomech.2013.11.009 24295566

[16] Eby, S. F. et al. Validation of shear wave elastography in skeletal muscle. *J. Biomech.* **46**, 2381–2387 (2013). 23953670 10.1016/j.jbiomech.2013.07.033 PMC3818126

[17] Nordez, A. & Hug, F. Muscle shear elastic modulus measured using supersonic shear imaging is highly related to muscle activity level. *J. Appl. Physiol.* **108**, 1389–1394 (2010). 20167669 10.1152/japplphysiol.01323.2009

[18] Lee, Y., Kim, M. & Lee, H. The measurement of stiffness for major muscles with shear wave elastography and myoton: A quantitative analysis study. *Diagnostics* **11**, 524 (2021). 33804273 10.3390/diagnostics11030524 PMC7999852

[19] Sasaki, K., Toyama, S. & Ishii, N. Length-force characteristics of in vivo human muscle reflected by supersonic shear imaging. *J. Appl. Physiol.* **117**, 153–162 (2014). 24876360 10.1152/japplphysiol.01058.2013

[20] Raiteri, B. J., Lauret, L. & Hahn, D. The force-length relation of the young adult human tibialis anterior. *PeerJ* **11**, e15693 (2023). 37461407 10.7717/peerj.15693 PMC10350298

[21] Zimmer, M., Kleiser, B., Marquetand, J. & Ateş, F. Shear wave elastography characterizes passive and active mechanical properties of biceps brachii muscle in vivo. *J. Mech. Behav. Biomed. Mater.* **137**, 105543 (2023). 36371993 10.1016/j.jmbbm.2022.105543

[22] Ateş, F., Marquetand, J. & Zimmer, M. Detecting age-related changes in skeletal muscle mechanics using ultrasound shear wave elastography. *Sci. Rep.* **13**, 20062 (2023). 37974024 10.1038/s41598-023-47468-z PMC10654699

[23] Ates, F. et al. Muscle shear elastic modulus is linearly related to muscle torque over the entire range of isometric contraction intensity. *J. Electromyogr. Kinesiol.* **25**, 703–708 (2015). 25956546 10.1016/j.jelekin.2015.02.005

[24] Le Sant, G., Ates, F., Brasseur, J. L. & Nordez, A. Elastography study of hamstring behaviors during passive stretching. *PLoS ONE* **10**, e0139272 (2015). 26418862 10.1371/journal.pone.0139272 PMC4587804

[25] Bouillard, K., Hug, F., Guével, A. & Nordez, A. Shear elastic modulus can be used to estimate an index of individual muscle force during a submaximal isometric fatiguing contraction. *J. Appl. Physiol.* **113**, 1353–1361 (2012). 22984244 10.1152/japplphysiol.00858.2012

[26] Maïsetti, O., Hug, F., Bouillard, K. & Nordez, A. Characterization of passive elastic properties of the human medial gastrocnemius muscle belly using supersonic shear imaging. *J. Biomech.* **45**, 978–984 (2012). 22326058 10.1016/j.jbiomech.2012.01.009

[27] Ates, F. et al. Passive stiffness of monoarticular lower leg muscles is influenced by knee joint angle. *Eur. J. Appl. Physiol.* **118**, 585–593 (2018). 29327169 10.1007/s00421-018-3798-y

[28] Bernabei, M., Lee, S. S. M., Perreault, E. J. & Sandercock, T. G. Axial stress determines the velocity of shear wave propagation in passive but not active muscles in vivo. *J. Appl. Physiol.* **134**, 941–950 (2023). 36861673 10.1152/japplphysiol.00125.2022 PMC10069958

[29] Zimmer, M. et al. In vivo assessment of shear wave propagation in pennate muscles using an automatic ultrasound probe alignment system. *IEEE Open J. Eng. Med. Biol.* **4**, 259–267 (2023). 38196975 10.1109/OJEMB.2023.3338090 PMC10776096

[30] Chino, K. & Takahashi, H. Influence of pennation angle on measurement of shear wave elastography: In vivo observation of shear wave propagation in human pennate muscle. *Physiol Meas* **39**, 115003 (2018). 30398162 10.1088/1361-6579/aae7e2

[31] Bernabei, X. M., Lee, S. S. M., Perreault, E. J. & Sandercock, T. G. Shear wave velocity is sensitive to changes in muscle stiffness that occur independently from changes in force. *J. Appl. Physiol.* **128**, 8–16 (2020). 31556833 10.1152/japplphysiol.00112.2019 PMC6985815

[32] Ruiz-Muñoz, M. & Cuesta-Vargas, A. I. Electromyography and sonomyography analysis of the tibialis anterior: A cross sectional study. *J. Foot Ankle Res.* **7**, 11 (2014). 24507748 10.1186/1757-1146-7-11 PMC3925007

[33] Byrne, C. A., O’Keeffe, D. T., Donnelly, A. E. & Lyons, G. M. Effect of walking speed changes on tibialis anterior EMG during healthy gait for FES envelope design in drop foot correction. *J. Electromyogr. Kinesiol.* **17**, 605–616 (2007). 16990012 10.1016/j.jelekin.2006.07.008

[34] Kimata, K. et al. Relationship between attachment site of tibialis anterior muscle and shape of tibia: Anatomical study of cadavers. *J. Foot Ankle Res.* **15**, 54 (2022). 35821059 10.1186/s13047-022-00559-y PMC9277928

[35] Gambelli, C. N. et al. The effect of tibialis anterior weakness on foot drop and toe clearance in patients with facioscapulohumeral dystrophy. *Clin. Biomech.* **102**, 105899 (2023). 10.1016/j.clinbiomech.2023.105899 36738507

[36] Imajo, Y., Nishida, N., Funaba, M., Suzuki, H. & Sakai, T. Factors associated with improvement in tibialis anterior weakness due to lumbar degenerative disease. *J. Orthop. Sci.* **29**, 734–740 (2024). 37149480 10.1016/j.jos.2023.03.011

[37] Hirata, K., Miyamoto-Mikami, E., Kanehisa, H. & Miyamoto, N. Muscle-specific acute changes in passive stiffness of human triceps surae after stretching. *Eur. J. Appl. Physiol.* **116**, 911–918 (2016). 26945574 10.1007/s00421-016-3349-3

[38] Raiteri, B. J., Cresswell, A. G. & Lichtwark, G. A. Muscle-Tendon length and force affect human tibialis anterior central aponeurosis stiffness in vivo. *Proc. Natl. Acad. Sci. U. S. A.* **115**, E3097–E3105 (2018). 29555756 10.1073/pnas.1712697115 PMC5889624

[39] Hug, F., Tucker, K., Gennisson, J. L., Tanter, M. & Nordez, A. Elastography for muscle biomechanics: Toward the estimation of individual muscle force. *Exerc. Sport Sci. Rev.* **43**, 125–133 (2015). 25906424 10.1249/JES.0000000000000049

[40] Koo, T. K., Guo, J. Y., Cohen, J. H. & Parker, K. J. Relationship between shear elastic modulus and passive muscle force: An ex-vivo study. *J. Biomech.* **46**, 2053–2059 (2013). 23769175 10.1016/j.jbiomech.2013.05.016

[41] Kaya, C. S., Yılmaz, E. O., Akdeniz-Doğan, Z. D. & Yucesoy, C. A. Long-term effects with potential clinical importance of botulinum toxin type-A on mechanics of muscles exposed. *Front. Bioeng. Biotechnol.* **8**, 738 (2020). 32695774 10.3389/fbioe.2020.00738 PMC7338794

[42] Yucesoy, C. A., Arikan, Ö. E. & Ates, F. BTX-A administration to the target muscle affects forces of all muscles within an intact compartment and epimuscular myofascial force transmission. *J. Biomech. Eng.* **134**(111002), 1–9 (2012). 10.1115/1.4007823 23387784

[43] Maganaris, C. N. Force-length characteristics of in vivo human skeletal muscle. *Acta Physiol. Scand.* **172**, 279–285 (2001). 11531649 10.1046/j.1365-201x.2001.00799.x

[44] Gordon, A. M., Huxley, A. F. & Julian, F. J. The variation in isometric tension with sarcomere length in vertebrate muscle fibres. *J. Physiol.* **184**, 170–192 (1966). 5921536 10.1113/jphysiol.1966.sp007909 PMC1357553

[45] Danesini, P. C., Heim, M., Tomalka, A., Siebert, T. & Ates, F. Endomysium determines active and passive force production in muscle fibers. *J. Biomech.* **168**, 112134 (2024). 38723428 10.1016/j.jbiomech.2024.112134

[46] Winters, T. M., Takahashi, M., Lieber, R. L. & Ward, S. R. Whole muscle length-tension relationships are accurately modeled as scaled sarcomeres in rabbit hindlimb muscles. *J. Biomech.* **44**, 109–115 (2011). 20889156 10.1016/j.jbiomech.2010.08.033 PMC3003754

[47] Whitehead, N. P., Gregory, J. E., Morgan, D. L. & Proske, U. Passive mechanical properties of the medial gastrocnemius muscle of the cat. *J. Physiol.* **536**, 893–903 (2001). 11691881 10.1111/j.1469-7793.2001.00893.x PMC2278914

[48] McNair, P. J., Hewson, D. J., Dombroski, E. & Stanley, S. N. Stiffness and passive peak force changes at the ankle joint: The effect of different joint angular velocities. *Clin. Biomech.* **17**, 536–540 (2002). 10.1016/s0268-0033(02)00062-1 12206945

[49] Hoang, P. D., Gorman, R. B., Todd, G., Gandevia, S. C. & Herbert, R. D. A new method for measuring passive length-tension properties of human gastrocnemius muscle in vivo. *J. Biomech.* **38**, 1333–1341 (2005). 15863118 10.1016/j.jbiomech.2004.05.046

[50] Nordez, A. et al. Improvements to Hoang et al.’s method for measuring passive length-tension properties of human gastrocnemius muscle in vivo. *J. Biomech.* **43**, 379–382 (2010). 19782365 10.1016/j.jbiomech.2009.07.034

[51] Rijkelijkhuizen, J. M., Meijer, H. J. M., Baan, G. C. & Huijing, P. A. Myofascial force transmission also occurs between antagonistic muscles located within opposite compartments of the rat lower hind limb. *J. Electromyogr. Kinesiol.* **17**, 690–697 (2007). 17383201 10.1016/j.jelekin.2007.02.004

[52] Ates, F. et al. Intramuscular pressure of tibialis anterior reflects ankle torque but does not follow joint angle-torque relationship. *Front. Physiol.* **9**, 22 (2018). 29416514 10.3389/fphys.2018.00022 PMC5787576

[53] Billot, M., Simoneau, E. M., Ballay, Y., Van Hoecke, J. & Martin, A. How the ankle joint angle alters the antagonist and agonist torques during maximal efforts in dorsi- and plantar flexion. *Scand. J. Med. Sci. Sports* **21**, e273–e281 (2011). 21392122 10.1111/j.1600-0838.2010.01278.x

[54] Koh, T. J. & Herzog, W. Evaluation of voluntary and elicited dorsiflexor torque-angle relationships. *J. Appl. Physiol.* **79**, 2007–2013 (1995). 8847267 10.1152/jappl.1995.79.6.2007

[55] Cudicio, A., Martinez-Valdes, E., Cogliati, M., Orizio, C. & Negro, F. The force-generation capacity of the tibialis anterior muscle at different muscle–tendon lengths depends on its motor unit contractile properties. *Eur. J. Appl. Physiol.* **122**, 317–330 (2022). 34677625 10.1007/s00421-021-04829-8 PMC8783895

[56] Rassier, D. E., MacIntosh, B. R. & Herzog, W. Length dependence of active force production in skeletal muscle. *J. Appl. Physiol.* **86**, 1445–1457 (1999). 10233103 10.1152/jappl.1999.86.5.1445

[57] Kirk, E. A. & Rice, C. L. Contractile function and motor unit firing rates of the human hamstrings. *J. Neurophysiol.* **117**, 243–250 (2017). 27784806 10.1152/jn.00620.2016 PMC5220116

[58] Hali, K., Zero, A. M. & Rice, C. L. Effect of ankle joint position on triceps surae contractile properties and motor unit discharge rates. *Physiol. Rep.* **8**, e14680 (2021). 33356017 10.14814/phy2.14680 PMC7757371

[59] Becker, R. & Awiszus, F. Physiological alterations of maximal voluntary quadriceps activation by changes of knee joint angle. *Muscle Nerve* **24**, 667–672 (2001). 11317277 10.1002/mus.1053

[60] Pasquet, B., Carpentier, A. & Duchateau, J. Change in muscle fascicle length influences the recruitment and discharge rate of motor units during isometric contractions. *J. Neurophysiol.* **94**, 3126–3133 (2005). 16014788 10.1152/jn.00537.2005

[61] Hodges, P. W., Pengel, L. H. M., Herbert, R. D. & Gandevia, S. C. Measurement of muscle contraction with ultrasound imaging. *Muscle Nerve* **27**, 682–692 (2003). 12766979 10.1002/mus.10375

[62] Manal, K., Roberts, D. P. & Buchanan, T. S. Can pennation angles be predicted from EMGs for the primary ankle plantar and dorsiflexors during isometric contractions?. *J. Biomech.* **41**, 2492–2497 (2008). 18579147 10.1016/j.jbiomech.2008.05.005 PMC2548308

[63] Farina, D., Merletti, R. & Enoka, R. M. The extraction of neural strategies from the surface EMG. *J. Appl. Physiol.* **96**, 1486–1495 (2004). 15016793 10.1152/japplphysiol.01070.2003

[64] Dideriksen, J. L. & Farina, D. Amplitude cancellation influences the association between frequency components in the neural drive to muscle and the rectified EMG signal. *PLoS Comput. Biol.* **15**, e1006985 (2019). 31050667 10.1371/journal.pcbi.1006985 PMC6519845

[65] Zimmer, M., Straub, L. F. & Ateş, F. Shear wave elastography reveals passive and active mechanics of triceps surae muscles in vivo: From shear modulus-ankle angle to stress-strain characteristics. *J. Appl. Physiol.* **1985**(138), 577–591 (2025). 10.1152/japplphysiol.00459.2024 39868629

[66] Dalton, B. et al. Central and peripheral neuromuscular fatigue following ramp and rapid maximal voluntary isometric contractions. *Front. Physiol.* **15**, 1434473 (2024). 39229620 10.3389/fphys.2024.1434473 PMC11368765

[67] Tomko, P. M., Colquhoun, R. J., Magrini, M. A., Muddle, T. W. D. & Jenkins, N. D. M. Global electromyographic signal characteristics depend on maximal isometric contraction method in the knee extensors. *J. Electromyogr. Kinesiol.* **42**, 111–116 (2018). 30015134 10.1016/j.jelekin.2018.07.002

[68] Tomko, P. M. et al. Maximal contraction methods influence the magnitude and reliability of global electromyographic signal characteristics. *J. Electromyogr. Kinesiol.* **48**, 121–127 (2019). 31344640 10.1016/j.jelekin.2019.07.002

[69] Oda, T. et al. In vivo length-force relationships on muscle fiber and muscle tendon complex in the tibialis anterior muscle. *Int. J. Sport Health Sci.* **3**, 245–252 (2005).

[70] Barrett, J. M., Malakoutian, M., Fels, S., Brown, S. H. M. & Oxland, T. R. Muscle short-range stiffness behaves like a maxwell element, not a spring: Implications for joint stability. *PLoS ONE* **19**, e0307977 (2024). 39141670 10.1371/journal.pone.0307977 PMC11324116

[71] Runge, C. F., Shupert, C. L., Horak, F. B. & Zajac, F. E. Ankle and hip postural strategies defined by joint torques. *Gait Posture* **10**, 161–170 (1999). 10502650 10.1016/s0966-6362(99)00032-6

[72] Marinho, H. V. R. et al. Myofascial force transmission in the lower limb: An in vivo experiment. *J. Biomech.* **63**, 55–60 (2017). 28838597 10.1016/j.jbiomech.2017.07.026

[73] Andrade, R. J., Lacourpaille, L., Freitas, S. R., Mcnair, P. J. & Nordez, A. Effects of hip and head position on ankle range of motion, ankle passive torque, and passive gastrocnemius tension. *Scand. J. Med. Sci. Sports* **26**, 41–47 (2016). 25676048 10.1111/sms.12406

[74] Arampatzis, A. et al. Differences between measured and resultant joint moments during isometric contractions at the ankle joint. *J. Biomech.* **38**, 885–892 (2005). 15713310 10.1016/j.jbiomech.2004.04.027

[75] Shin, H. J., Kim, M. J., Kim, H. Y., Roh, Y. H. & Lee, M. J. Comparison of shear wave velocities on ultrasound elastography between different machines, transducers, and acquisition depths: A phantom study. *Eur. Radiol.* **26**, 3361–3367 (2016). 26815368 10.1007/s00330-016-4212-y

[76] Chen, Q., Shi, B., Zheng, Y. & Hu, X. Analysis of influencing factors of shear wave elastography of the superficial tissue: A phantom study. *Front. Med.* **9**, 2249 (2022). 10.3389/fmed.2022.943844 PMC9393305 36004380

[77] Raiteri, B. J., Hug, F., Cresswell, A. G. & Lichtwark, G. A. Quantification of muscle co-contraction using supersonic shear wave imaging. *J. Biomech.* **49**, 493–495 (2016). 26776929 10.1016/j.jbiomech.2015.12.039

[78] Ito, M., Kawakami, Y., Ichinose, Y., Fukashiro, S. & Fukunaga, T. Nonisometric behavior of fascicles during isometric contractions of a human muscle. *J. Appl. Physiol.* **85**, 1230–1235 (1998). 9760310 10.1152/jappl.1998.85.4.1230

[79] Powell, P. L., Roy, R. R., Kanim, P., Bello, M. A. & Edgerton, V. R. Predictability of skeletal muscle tension from architectural determinations in guinea pig hindlimbs. *J. Appl. Physiol.* **57**, 1715–1721 (1984). 6511546 10.1152/jappl.1984.57.6.1715

[80] Fukunaga, T., Roy, R. R., Shellock, F. G., Hodgson, J. A. & Edgerton, V. R. Specific tension of human plantar flexors and dorsiflexors. *J. Appl. Physiol.* **80**, 158–165 (1996). 8847297 10.1152/jappl.1996.80.1.158

[81] Sahrmann, A. S., Vosse, L., Siebert, T., Handsfield, G. G. & Röhrle, O. Determination of muscle shape deformations of the tibialis anterior during dynamic contractions using 3D ultrasound. *Front. Bioeng. Biotechnol.* **12**, 1388907 (2024). 38903187 10.3389/fbioe.2024.1388907 PMC11188672

[82] Rugg, S. G., Gregor, R. J., Mandelbaum, B. R. & Chiu, L. In vivo moment arm calculations at the ankle using magnetic resonance imaging (MRI). *J. Biomech.* **23**, 495–501 (1990). 2373722 10.1016/0021-9290(90)90305-m

[83] Tsaopoulos, D. E., Maganaris, C. N. & Baltzopoulos, V. Can the patellar tendon moment arm be predicted from anthropometric measurements?. *J. Biomech.* **40**, 645–651 (2007). 16542664 10.1016/j.jbiomech.2006.01.022

[84] Hermens, H. J., Freriks, B., Disselhorst-Klug, C. & Rau, G. Development of recommendations for SEMG sensors and sensor placement procedures. *J. Electromyogr. Kinesiol.* **10**, 361–374 (2000). 11018445 10.1016/s1050-6411(00)00027-4

[85] Crutison, J., Sun, M. & Royston, T. J. The combined importance of finite dimensions, anisotropy, and pre-stress in acoustoelastography. *J. Acoust. Soc. Am.* **151**, 2403 (2022). 35461517 10.1121/10.0010110 PMC8993425

[86] Drazan, J. F., Hullfish, T. J. & Baxter, J. R. An automatic fascicle tracking algorithm quantifying gastrocnemius architecture during maximal effort contractions. *PeerJ* **2019**, e7120 (2019). 10.7717/peerj.7120 PMC6611451 31304054

[87] Brand, R. A., Pedersen, D. R. & Friederich, J. A. The sensitivity of muscle force predictions to changes in physiologic cross-sectional area. *J. Biomech.* **19**, 589–596 (1986). 3771581 10.1016/0021-9290(86)90164-8

[88] Maganaris, C. N. & Paul, J. P. Load-elongation characteristics of in vivo human tendon and aponeurosis. *J. Exp. Biol.* **203**, 751–756 (2000). 10648216 10.1242/jeb.203.4.751

[89] Fukunaga, T. et al. Physiological cross-sectional area of human leg muscles based on magnetic resonance imaging. *J. Orthop. Res.* **10**, 928–934 (1992). 1403308 10.1002/jor.1100100623

[90] Vanmechelen, I. M., Shortland, A. P. & Noble, J. J. Lower limb muscle volume estimation from maximum cross-sectional area and muscle length in cerebral palsy and typically developing individuals. *Clin. Biomech.* **51**, 40–44 (2018). 10.1016/j.clinbiomech.2017.11.004 29179032

