# Supplementary material for: In vivo tibialis anterior muscle mechanics through force estimation using ankle joint moment and shear wave elastography
Source: Sci Rep. 2025 Sep 12;15:32461. doi: 10.1038/s41598-025-18292-4 (PMC12432256; doi:10.1038/s41598-025-18292-4)
Supplement: Supplementary file 1 — Supplementary Information 1. [file 41598_2025_18292_MOESM1_ESM.pdf]

In vivo tibialis anterior muscle mechanics through force estimation using ankle joint moment and shear wave elastography

Supplementary 1: Fascicle length and pennation angle analysis

The tibialis anterior (TA) muscle architecture was evaluated using B-mode ultrasound videos recorded in two states: rest (passive state) and during maximum voluntary contraction (MVC). For each participant, fascicle lengths and pennation angles were calculated for each tested ankle angle, for both the superficial and deep compartments of the TA.

An adapted version of a semi-automatic fascicle tracking algorithm[1] was used to analyze each video. In each trial, three fascicles per compartment were selected, and their average values were used in subsequent analyses. Pennation angles were calculated as the angle between the fascicles in each compartment and the central aponeurosis.

In addition to assessing muscle architecture in different conditions (passive state and MVC), the compartment-specific fascicle lengths and pennation angles recorded during MVC were used to estimate TA force and the relative contributions of each compartment.

The following research questions were addressed to explore potential compartmental differences and the effects of muscle activation:

> ***Compartmental Differences*** Do fascicle lengths and/or pennation angles differ between the superficial and deep compartments in the passive state and during MVC? If so, are these differences dependent on ankle angle?
>
> ***Effects of Muscle Activation*** Do fascicle lengths and/or pennation angles differ between conditions (i.e., passive state vs. MVC) in the superficial and deep compartments? If so, are these differences ankle-angle specific?

Figure 1 illustrates fascicle length and pennation angles in the superficial and deep compartments of the TA, with detailed statistical findings provided below.

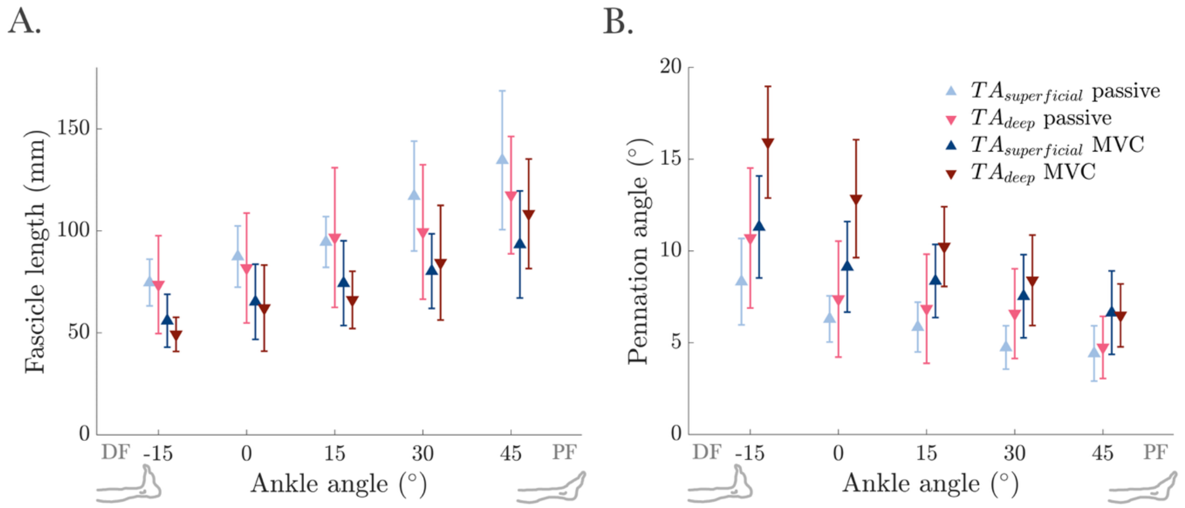

Figure 1. (A) Fascicle length and (B) Pennation angles in the superficial and deep compartments of the tibialis anterior (TA) muscle as functions of ankle angles studied. Data are presented for both the passive state and maximum voluntary contraction (MVC). Error bars visualize standard deviations. DF: dorsiflexion, PF: plantar flexion.

Fascicle Length

Fascicle lengths from the superficial and deep compartments of the TA muscle, measured at rest and during MVC as a function of studied ankle angles, are shown in Table 1. Performing a 2-way repeated measures analysis of variance (ANOVA), the following findings were revealed:

Table 1. Fascicle lengths from the superficial and deep compartments of the TA muscle, at rest and during MVC as functions of ankle angles studied. Data is given as mean ± standard deviation.

	Ankle angle				
	-15°	0°	15°	30°	45°
Passive state, superficial compartment	74.68 ± 11.45	87.40 ± 15.02	94.57 ± 12.43	117.07 ± 26.93	134.64 ± 34.05
Passive state, deep compartment	73.63 ± 24.00	81.78 ± 26.95	96.73 ± 34.25	99.46 ± 32.97	117.52 ± 28.77
MVC, superficial compartment	55.93 ± 12.97	65.22 ± 18.44	74.35 ± 20.79	80.26 ± 18.30	93.35 ± 26.24
MVC, deep compartment	49.25 ± 8.38	62.10 ± 21.11	66.15 ± 14.04	84.38 ± 28.11	108.38 ± 26.86

Compartmental Differences The fascicle lengths from the superficial and deep compartments were not significantly different in either the passive or MVC states (Table 2).

Table 2. Two-way repeated measures ANOVA results for fascicle length differences between constitutive compartments of the TA muscle at rest and during MVC. Reported as F(d1, d2) = F-value, p = p-value, where d1 and d2 are the numerator and denominator degrees of freedom.

**Passive state: The effects of ankle angle (Factor 1) and compartment (Factor 2)**	
Factor 1	$F(4, 36) = 31.57$, $p < 0.001$<br>Post-hoc analysis for Factor 1<br>-15° vs. 0°: $F(1, 36) = 29.47$, $p = 0.004$<br>-15° vs. 15°: $F(1, 36) = 19.48$, $p = 0.017$<br>-15° vs. 30°: $F(1, 36) = 37.61$, $p = 0.002$<br>-15° vs. 45°: $F(1, 36) = 55.88$, $p < 0.001$<br>0° vs. 30°: $F(1, 36) = 19.00$, $p = 0.018$<br>0° vs. 45°: $F(1, 36) = 40.81$, $p = 0.001$<br>15° vs. 45°: $F(1, 36) = 30.06$, $p = 0.004$<br>30° vs. 45°: $F(1, 36) = 36.45$, $p = 0.002$
Factor 2	$F(1, 9) = 4.52$, $p = 0.062$
Interaction	$F(4, 36) = 2.95$, $p = 0.033$<br>Post-hoc for Factor 1 with Factor 2<br>*superficial*: -15° vs. 0°: $F(1, 36) = 28.38$, $p = 0.005$<br>*superficial*: -15° vs. 15°: $F(1, 36) = 21.34$, $p = 0.013$<br>*superficial*: -15° vs. 30°: $F(1, 36) = 28.52$, $p = 0.005$<br>*superficial*: -15° vs. 45°: $F(1, 36) = 46.11$, $p = 0.001$<br>*superficial*: 0° vs. 30°: $F(1, 36) = 16.92$, $p = 0.026$<br>*superficial*: 0° vs. 45°: $F(1, 36) = 34.33$, $p = 0.002$<br>*superficial*: 15° vs. 30°: $F(1, 36) = 19.64$, $p = 0.016$<br>*superficial*: 15° vs. 45°: $F(1, 36) = 36.87$, $p = 0.002$<br>*superficial*: 30° vs. 45°: $F(1, 36) = 18.94$, $p = 0.018$<br>*deep*: -15° vs. 30°: $F(1, 36) = 20.18$, $p = 0.015$<br>*deep*: -15° vs. 45°: $F(1, 36) = 39.08$, $p = 0.001$<br>*deep*: 0° vs. 45°: $F(1, 36) = 22.51$, $p = 0.011$<br>Post-hoc for Factor 2 with Factor 1 (*superficial* vs. *deep*)<br>45°: $F(1, 36) = 10.27$, $p = 0.011$
**MVC: The effects of ankle angle (Factor 1) and compartment (Factor 2)**	
Factor 1	$F(4, 28) = 35.02$, $p < 0.001$<br>Post-hoc analysis for Factor 1<br>-15° vs. 15°: $F(1, 28) = 70.27$, $p = 0.001$<br>-15° vs. 30°: $F(1, 28) = 53.07$, $p = 0.002$<br>-15° vs. 45°: $F(1, 28) = 52.45$, $p = 0.002$<br>0° vs. 30°: $F(1, 28) = 26.51$, $p = 0.013$<br>0° vs. 45°: $F(1, 28) = 27.80$, $p = 0.012$<br>15° vs. 30°: $F(1, 28) = 24.32$, $p = 0.017$<br>15° vs. 45°: $F(1, 28) = 33.39$, $p = 0.007$<br>30° vs. 45°: $F(1, 28) = 26.11$, $p = 0.014$
Factor 2	$F(1, 7) = 4.39$, $p = 0.074$
Interaction	$F(4, 28) = 0.79$, $p = 0.543$

Effects of Muscle Activation Fascicle lengths were significantly shorter during MVC compared to the passive state (Table 3).

For the superficial compartment, fascicle lengths were shorter during MVC compared to the passive state by 16.18 mm (25.11%), 21.81 mm (25.38%), 20.22 mm (21.38%), 36.81 mm (31.44%), and 41.30 mm (30.67%) at ankle angles of -15°, 0°, 15°, 30°, and 45°, respectively.

For the deep compartment, fascicles were, on average, 19.77 mm (22.34%) shorter during MVC compared to the passive state.

Table 3. Two-way repeated measures ANOVA results for fascicle length differences between testing conditions for the constitutive compartments of the TA muscle. Reported as F(d1, d2) = F-value, p = p-value, where d1 and d2 are the numerator and denominator degrees of freedom.

**Superficial compartment: The effects of ankle angle (Factor 1) and testing condition (Factor 2)**	
Factor 1	F(4, 44) = 30.07, p < 0.001<br>Post-hoc analysis for Factor 1<br>-15° vs. 0°: F(1, 44) = 21.53, p = 0.007<br>-15° vs. 15°: F(1, 44) = 45.54, p < 0.001<br>-15° vs. 30°: F(1, 44) = 43.75, p < 0.001<br>-15° vs. 45°: F(1, 44) = 46.42, p < 0.001<br>0° vs. 30°: F(1, 44) = 23.68, p = 0.005<br>0° vs. 45°: F(1, 44) = 30.77, p = 0.002<br>15° vs. 45°: F(1, 44) = 20.82, p = 0.008<br>30° vs. 45°: F(1, 44) = 26.29, p = 0.003
Factor 2	F(1, 11) = 50.57, p < 0.001
Interaction	F(4, 44) = 4.48, p = 0.004<br>Post-hoc for Factor 1 with Factor 2<br>*passive*: -15° vs. 0°: F(1, 44) = 34.48, p = 0.001<br>*passive*: -15° vs. 15°: F(1, 44) = 32.75, p = 0.001<br>*passive*: -15° vs. 30°: F(1, 44) = 37.20, p = 0.001<br>*passive*: -15° vs. 45°: F(1, 44) = 48.40, p < 0.001<br>*passive*: 0° vs. 30°: F(1, 44) = 22.79, p = 0.006<br>*passive*: 0° vs. 45°: F(1, 44) = 36.55, p = 0.001<br>*passive*: 15° vs. 30°: F(1, 44) = 16.26, p = 0.020<br>*passive*: 15° vs. 45°: F(1, 44) = 24.67, p = 0.004<br>*passive*: 30° vs. 45°: F(1, 44) = 14.75, p = 0.027<br>*MVC*: -15° vs. 15°: F(1, 44) = 14.68, p = 0.028<br>*MVC*: -15° vs. 30°: F(1, 44) = 18.22, p = 0.013<br>*MVC*: -15° vs. 45°: F(1, 44) = 19.90, p = 0.010<br>Post-hoc for Factor 2 with Factor 1 (*passive* vs. *MVC*)<br>-15°: F(1, 44) = 19.24, p = 0.001<br>0°: F(1, 44) = 17.61, p = 0.001<br>15°: F(1, 44) = 12.75, p = 0.004<br>30°: F(1, 44) = 35.97, p < 0.001<br>45°: F(1, 44) = 23.45, p = 0.001
**Deep compartment: The effects of ankle angle (Factor 1) and testing condition (Factor 2)**	
Factor 1	F(4, 28) = 36.68, p < 0.001<br>Post-hoc analysis for Factor 1<br>-15° vs. 0°: F(1, 28) = 37.28, p = 0.005<br>-15° vs. 15°: F(1, 28) = 22.74, p = 0.020<br>-15° vs. 30°: F(1, 28) = 41.71, p = 0.003<br>-15° vs. 45°: F(1, 28) = 77.83, p < 0.001

	0° vs. 30°: F(1, 28) = 24.20, p = 0.017<br>0° vs. 45°: F(1, 28) = 49.23, p = 0.002<br>15° vs. 45°: F(1, 28) = 23.88, p = 0.018<br>30° vs. 45°: F(1, 28) = 35.00, p = 0.006
Factor 2	F(1, 7) = 12.86, p = 0.009
Interaction	F(4, 28) = 1.05, p = 0.398

Pennation Angle

Pennation angles for fascicles from the superficial and deep compartments of the TA muscle, at rest and during MVC as functions of ankle angles studied are shown in Table 4. Performing 2-way repeated measures ANOVA tests, the following findings were revealed:

Table 4. Pennation angles for fascicles from the superficial and deep compartments of the TA muscle, at rest and during MVC as functions of ankle angles studied. Data is given as mean ± standard deviation.

	Ankle angle				
	-15°	0°	15°	30°	45°
Passive state, superficial compartment	8.33 ± 2.35	6.29 ± 1.26	5.85 ± 1.36	4.74 ± 1.18	4.41 ± 1.51
Passive state, deep compartment	10.70 ± 3.81	7.38 ± 3.16	6.85 ± 2.97	6.58 ± 2.45	4.74 ± 1.69
MVC, superficial compartment	11.31 ± 2.78	9.13 ± 2.47	8.36 ± 1.99	7.53 ± 2.27	6.64 ± 2.27
MVC, deep compartment	15.92 ± 3.04	12.85 ± 3.21	10.24 ± 2.17	3.40 ± 2.47	6.49 ± 1.71

Compartmental Differences The pennation angles of the superficial and deep compartments were significantly different in both the passive state and MVC (Table 5).

> **In the passive state**, pennation angles in the deep compartment were significantly larger than those in the superficial compartment at -15° and 30°, with differences of 3.36° (28.55%) and 2.05° (38.92%), respectively.
>
> **During MVC**, pennation angles in the deep compartment were significantly larger than those in the superficial compartment at -15°, 0°, and 15°, with differences of 5.25° (40.84%), 3.68° (40.69%), and 2.40° (22.43%), respectively.

Table 5. Two-way repeated measures ANOVA results for pennation angle differences between constitutive compartments of the TA muscle at rest and during MVC. Reported as F(d1, d2) = F-value, p = p-value, where d1 and d2 are the numerator and denominator degrees of freedom.

**Passive state: The effects of ankle angle (Factor 1) and compartment (Factor 2)**	
Factor 1	F(4, 36) = 54.50, p < 0.001<br>Post-hoc analysis for Factor 1<br>-15° vs. 0°: F(1, 36) = 57.02, p < 0.001<br>-15° vs. 15°: F(1, 36) = 53.82, p < 0.001<br>-15° vs. 30°: F(1, 36) = 75.70, p < 0.001

	-15° vs. 45°: $F(1, 36) = 87.05$, $p < 0.001$<br>0° vs. 30°: $F(1, 36) = 37.15$, $p = 0.002$<br>0° vs. 45°: $F(1, 36) = 65.78$, $p < 0.001$<br>15° vs. 45°: $F(1, 36) = 22.11$, $p = 0.011$<br>30° vs. 45°: $F(1, 36) = 16.90$, $p = 0.026$
Factor 2	$F(1, 9) = 5.23$, $p = 0.048$
Interaction	$F(4, 36) = 2.66$, $p = 0.048$<br>Post-hoc for Factor 1 with Factor 2<br>*superficial*: -15° vs. 15°: $F(1, 36) = 28.12$, $p = 0.005$<br>*superficial*: -15° vs. 30°: $F(1, 36) = 52.49$, $p < 0.001$<br>*superficial*: -15° vs. 45°: $F(1, 36) = 75.14$, $p < 0.001$<br>*superficial*: 0° vs. 45°: $F(1, 36) = 15.40$, $p = 0.035$<br>*superficial*: 15° vs. 30°: $F(1, 36) = 36.43$, $p = 0.002$<br>*superficial*: 15° vs. 45°: $F(1, 36) = 52.03$, $p = 0.001$<br>*deep*: -15° vs. 0°: $F(1, 36) = 36.68$, $p = 0.002$<br>*deep*: -15° vs. 15°: $F(1, 36) = 29.93$, $p = 0.004$<br>*deep*: -15° vs. 30°: $F(1, 36) = 30.42$, $p = 0.004$<br>*deep*: -15° vs. 45°: $F(1, 36) = 33.07$, $p = 0.003$<br>*deep*: 0° vs. 45°: $F(1, 36) = 17.96$, $p = 0.022$<br>Post-hoc for Factor 2 with Factor 1 (*superficial* vs. *deep*)<br>-15°: $F(1, 36) = 5.96$, $p = 0.037$<br>30°: $F(1, 36) = 8.55$, $p = 0.017$
**MVC: The effects of ankle angle (Factor 1) and compartment (Factor 2)**	
Factor 1	$F(4, 36) = 102.36$, $p < 0.001$<br>Post-hoc analysis for Factor 1<br>-15° vs. 0°: $F(1, 36) = 75.44$, $p < 0.001$<br>-15° vs. 15°: $F(1, 36) = 153.73$, $p < 0.001$<br>-15° vs. 30°: $F(1, 36) = 130.47$, $p < 0.001$<br>-15° vs. 45°: $F(1, 36) = 386.42$, $p < 0.001$<br>0° vs. 15°: $F(1, 36) = 23.69$, $p = 0.009$<br>0° vs. 30°: $F(1, 36) = 35.25$, $p = 0.002$<br>0° vs. 45°: $F(1, 36) = 79.47$, $p < 0.001$<br>15° vs. 30°: $F(1, 36) = 29.19$, $p = 0.004$<br>15° vs. 45°: $F(1, 36) = 144.34$, $p < 0.001$<br>30° vs. 45°: $F(1, 36) = 15.29$, $p = 0.036$
Factor 2	$F(1, 9) = 14.80$, $p = 0.004$
Interaction	$F(4, 36) = 7.08$, $p < 0.001$<br>Post-hoc for Factor 1 with Factor 2<br>*superficial*: -15° vs. 0°: $F(1, 36) = 28.83$, $p = 0.005$<br>*superficial*: -15° vs. 15°: $F(1, 36) = 51.06$, $p = 0.001$<br>*superficial*: -15° vs. 30°: $F(1, 36) = 34.39$, $p = 0.002$<br>*superficial*: -15° vs. 45°: $F(1, 36) = 30.86$, $p = 0.004$<br>*deep*: -15° vs. 0°: $F(1, 36) = 32.90$, $p = 0.003$<br>*deep*: -15° vs. 15°: $F(1, 36) = 159.41$, $p < 0.001$<br>*deep*: -15° vs. 30°: $F(1, 36) = 90.99$, $p < 0.001$<br>*deep*: -15° vs. 45°: $F(1, 36) = 186.85$, $p < 0.001$<br>*deep*: 0° vs. 15°: $F(1, 36) = 25.24$, $p = 0.007$<br>*deep*: 0° vs. 30°: $F(1, 36) = 45.60$, $p = 0.001$<br>*deep*: 0° vs. 45°: $F(1, 36) = 78.36$, $p < 0.001$<br>*deep*: 15° vs. 30°: $F(1, 36) = 15.31$, $p = 0.035$

	deep: 15° vs. 45°: F(1, 36) = 57.67, p < 0.001<br>Post-hoc for Factor 2 with Factor 1 (*superficial* vs. *deep*)<br>-15°: F(1, 36) = 16.47, p = 0.003<br>0°: F(1, 36) = 9.70, p = 0.012<br>15°: F(1, 36) = 10.00, p = 0.012

Effects of Muscle Activation The testing conditions (passive vs. MVC) significantly affected pennation angles of both compartments, with higher pennation angles observed during MVC compared to the passive state (Table 6).

For the superficial compartment, pennation angles were on average 2.67° (46.62%) higher during MVC compared to the passive state.

For the deep compartment, pennation angles were higher during MVC compared to the passive state, with differences of 5.76° (48.78%), 5.56° (74.22%), 3.73° (49.45%), 1.95° (27.57%), and 1.64° (36.77%) at -15°, 0°, 15°, 30°, and 45° ankle angles, respectively.

Table 6. Two-way repeated measures ANOVA results for pennation angle differences between testing conditions for the constitutive compartments of the TA muscle. Reported as F(d1, d2) = F-value, p = p-value, where d1 and d2 are the numerator and denominator degrees of freedom.

**Superficial compartment: The effects of ankle angle (Factor 1) and testing condition (Factor 2)**	
Factor 1	F(4, 44) = 31.52, p < 0.001<br>Post-hoc analysis for Factor 1<br>-15° vs. 0°: F(1, 44) = 20.12, p = 0.009<br>-15° vs. 15°: F(1, 44) = 54.35, p < 0.001<br>-15° vs. 30°: F(1, 44) = 84.32, p < 0.001<br>-15° vs. 45°: F(1, 44) = 65.09, p < 0.001<br>0° vs. 45°: F(1, 44) = 13.76, p = 0.034<br>15° vs. 30°: F(1, 44) = 21.39, p = 0.007<br>15° vs. 45°: F(1, 44) = 23.74, p = 0.005
Factor 2	F(1, 11) = 29.90, p < 0.001
Interaction	F(4, 44) = 0.31, p = 0.868
**Deep compartment: The effects of ankle angle (Factor 1) and testing condition (Factor 2)**	
Factor 1	F(4, 36) = 76.71, p < 0.001<br>Post-hoc analysis for Factor 1<br>-15° vs. 0°: F(1, 36) = 63.07, p < 0.001<br>-15° vs. 15°: F(1, 36) = 139.39, p < 0.001<br>-15° vs. 30°: F(1, 36) = 131.49, p < 0.001<br>-15° vs. 45°: F(1, 36) = 165.25, p < 0.001<br>0° vs. 30°: F(1, 36) = 33.59, p = 0.003<br>0° vs. 45°: F(1, 36) = 78.30, p < 0.001<br>15° vs. 45°: F(1, 36) = 28.76, p = 0.005
Factor 2	F(1, 9) = 58.57, p < 0.001
Interaction	F(4, 36) = 8.90, p < 0.001<br>Post-hoc for Factor 1 with Factor 2<br>*passive*: -15° vs. 0°: F(1, 36) = 36.68, p = 0.002<br>*passive*: -15° vs. 15°: F(1, 36) = 29.93, p = 0.004<br>*passive*: -15° vs. 30°: F(1, 36) = 30.42, p = 0.004<br>*passive*: -15° vs. 45°: F(1, 36) = 33.07, p = 0.003<br>*passive*: 0° vs. 45°: F(1, 36) = 17.96, p = 0.022

	MVC: -15° vs. 0°: $F(1, 36) = 32.90$, $p = 0.003$
	MVC: -15° vs. 15°: $F(1, 36) = 159.41$, $p < 0.001$
	MVC: -15° vs. 30°: $F(1, 36) = 90.99$, $p < 0.001$
	MVC: -15° vs. 45°: $F(1, 36) = 186.85$, $p < 0.001$
	MVC: 0° vs. 15°: $F(1, 36) = 25.24$, $p = 0.007$
	MVC: 0° vs. 30°: $F(1, 36) = 45.60$, $p = 0.001$
	MVC: 0° vs. 45°: $F(1, 36) = 78.36$, $p < 0.001$
	MVC: 15° vs. 30°: $F(1, 36) = 15.31$, $p = 0.035$
	MVC: 15° vs. 45°: $F(1, 36) = 57.67$, $p < 0.001$
	Post-hoc for Factor 2 with Factor 1 (*passive* vs. *MVC*)
	-15°: $F(1, 36) = 24.44$, $p = 0.001$
	0°: $F(1, 36) = 57.75$, $p < 0.001$
	15°: $F(1, 36) = 24.13$, $p = 0.001$
	30°: $F(1, 36) = 17.33$, $p = 0.002$
	45°: $F(1, 36) = 14.53$, $p = 0.004$

References

1. Drazan, J. F., Hullfish, T. J. & Baxter, J. R. An automatic fascicle tracking algorithm quantifying gastrocnemius architecture during maximal effort contractions. *PeerJ* **2019**, e7120 (2019).
