# Supplementary material for: In vivo tibialis anterior muscle mechanics through force estimation using ankle joint moment and shear wave elastography
Source: Sci Rep. 2025 Sep 12;15:32461. doi: 10.1038/s41598-025-18292-4 (PMC12432256; doi:10.1038/s41598-025-18292-4)
Supplement: Supplementary file 2 — Supplementary Information 2. [file 41598_2025_18292_MOESM2_ESM.pdf]

In vivo tibialis anterior muscle mechanics through force estimation using ankle joint moment and shear wave elastography

Supplementary 2: EMG characteristics of the muscles studied

During the experiment, the electrical activity of the tibialis anterior muscle, as well as the extensor digitorum longus in the same compartment, and muscles from other compartments, including the peroneus longus in the lateral compartment, and the gastrocnemius medialis, gastrocnemius lateralis, and soleus in the posterior compartment, was monitored. Repeated measures analysis of variance (ANOVA) tests revealed the following findings for the EMG recorded during maximum voluntary contraction (MVC) and submaximal ramp contractions:

Maximum Voluntary Contraction

Ankle angle had a significant effect on the EMG amplitudes of the tested muscles during MVC (Table 1).

Table 1. One-way repeated measures ANOVA and post-hoc analysis results showing the effects of ankle angle on EMG amplitude during MVC. Reported as F(d1, d2) = F-value, p = p-value, where d1 and d2 are the numerator and denominator degrees of freedom. Pairwise differences are expressed as the percentage change in EMG amplitude at the second ankle angle relative to the first.

**Tibialis anterior**	
$F(4, 52) = 19.68$, $p < 0.001$	
	-15° vs. 0°: $F(1, 52) = 23.05$, $p = 0.003$, difference: -12.98%
	-15° vs. 15°: $F(1, 52) = 29.73$, $p = 0.001$, difference: -16.75%
	-15° vs. 30°: $F(1, 52) = 29.77$, $p = 0.001$, difference: -26.08%
	-15° vs. 45°: $F(1, 52) = 27.38$, $p = 0.002$, difference: -36.69%
	0° vs. 30°: $F(1, 52) = 11.59$, $p = 0.047$, difference: -14.87%
	0° vs. 45°: $F(1, 52) = 14.89$, $p = 0.020$, difference: -26.90%
	15° vs. 45°: $F(1, 52) = 14.49$, $p = 0.022$, difference: -23.93%
**Extensor digitorum longus**	
$F(4, 52) = 11.80$, $p < 0.001$	
	-15° vs. 45°: $F(1, 52) = 21.56$, $p = 0.005$, difference: -27.28%
	0° vs. 45°: $F(1, 52) = 16.33$, $p = 0.014$, difference: -23.79%
	15° vs. 30°: $F(1, 52) = 12.32$, $p = 0.038$, difference: -8.34%
	15° vs. 45°: $F(1, 52) = 12.92$, $p = 0.033$, difference: -23.20%
**Gastrocnemius medialis**	
$F(4, 52) = 3.33$, $p = 0.017$	
	0° vs. 15°: $F(1, 52) = 19.90$, $p = 0.006$, difference: 14.81%
	0° vs. 30°: $F(1, 52) = 15.76$, $p = 0.016$, difference: 19.83%
**Gastrocnemius lateralis**	
$F(4, 52) = 1.02$, $p = 0.407$	
**Soleus**	
$F(4, 48) = 5.08$, $p = 0.002$	

	-15° vs. 15°: F(1, 48) = 33.02, p = 0.001, difference: 31.98%
	-15° vs. 30°: F(1, 48) = 22.91, p = 0.004, difference: 39.83%
	0° vs. 15°: F(1, 48) = 19.06, p = 0.009, difference: 18.08%
	0° vs. 30°: F(1, 48) = 17.95, p = 0.012, difference: 24.57%
**Peroneus longus**	
F(4, 52) = 4.98, p = 0.002	
	-15° vs. 15°: F(1, 52) = 16.72, p = 0.013, difference: 42.09%
	-15° vs. 30°: F(1, 52) = 21.82, p = 0.004, difference: 55.75%

Submaximal Ramp Contractions

Significant differences in the normalized EMG amplitudes of various muscles tested were observed depending on the ankle angle and contraction intensity (i.e., 25%, 50%, and 75% of MVC moment) (Table 2).

Table 2. Two-way repeated measures ANOVA and post-hoc analysis results showing the effects of ankle angle (Factor 1) and contraction intensity (Factor 2) on the normalized EMG amplitude of tested muscles during submaximal ramp contractions. Reported as F(d1, d2) = F-value, p = p-value, where d1 and d2 are the numerator and denominator degrees of freedom.

**Tibialis anterior**	
Factor 1	F(4, 52) = 26.54, p < 0.001<br>Post-hoc analysis for Factor 1<br>-15° vs. 30°: F(1, 52) = 16.67, p = 0.013<br>-15° vs. 45°: F(1, 52) = 57.02, p < 0.001<br>0° vs. 30°: F(1, 52) = 15.16, p = 0.018<br>0° vs. 45°: F(1, 52) = 49.34, p < 0.001<br>15° vs. 30°: F(1, 52) = 12.19, p = 0.040<br>15° vs. 45°: F(1, 52) = 51.54, p < 0.001<br>30° vs. 45°F(1, 52) = 17.11, p = 0.012
Factor 2	F(2, 26) = 501.89, p < 0.001<br>Post-hoc analysis for Factor 2<br>25% vs. 50%: F(1, 26) = 189.44, p < 0.001<br>25% vs. 75%: F(1, 26) = 677.56, p < 0.001<br>50% vs. 75%: F(1, 26) = 600.15, p < 0.001
Interaction	F(8, 104) = 5.45, p < 0.001<br>Post-hoc for Factor 1 with Factor 2<br>*25%*: -15° vs 45°: F(1, 104) = 21.70, p = 0.004<br>*25%*: 0° vs 45°: F(1, 104) = 41.24, p < 0.001<br>*25%*: 15° vs 45°: F(1, 104) = 40.96, p < 0.001<br>*25%*: 30° vs 45°: F(1, 104) = 25.57, p = 0.002<br>*50%*: -15° vs 30°: F(1, 104) = 21.94, p = 0.004<br>*50%*: -15° vs 45°: F(1, 104) = 101.09, p < 0.001<br>*50%*: 0° vs 30°: F(1, 104) = 20.84, p = 0.005<br>*50%*: 0° vs 45°: F(1, 104) = 66.65, p < 0.001<br>*50%*: 15° vs 30°: F(1, 104) = 18.49, p = 0.009<br>*50%*: 15° vs 45°: F(1, 104) = 66.90, p < 0.001<br>*50%*: 30° vs 45°: F(1, 104) = 13.63, p = 0.027<br>*75%*: -15° vs 30°: F(1, 104) = 18.94, p = 0.008<br>*75%*: -15° vs 45°: F(1, 104) = 13.85, p = 0.026<br>*75%*: 15° vs 45°: F(1, 104) = 12.77, p = 0.034

	Post-hoc for Factor 2 with Factor 1
	-15°: *25%* vs. *50%* F(1, 104) = 62.44, p < 0.001
	-15°: *25%* vs. *75%* F(1, 104) = 346.72, p < 0.001
	-15°: *50%* vs. *75%* F(1, 104) = 388.57, p < 0.001
	0°: *25%* vs. *50%* F(1, 104) = 129.05, p < 0.001
	0°: *25%* vs. *75%* F(1, 104) = 383.00, p < 0.001
	0°: *50%* vs. *75%* F(1, 104) = 247.52, p < 0.001
	15°: *25%* vs. *50%* F(1, 104) = 146.51, p < 0.001
	15°: *25%* vs. *75%* F(1, 104) = 372.11, p < 0.001
	15°: *50%* vs. *75%* F(1, 104) = 206.13, p < 0.001
	30°: *25%* vs. *50%* F(1, 104) = 237.74, p < 0.001
	30°: *25%* vs. *75%* F(1, 104) = 449.36, p < 0.001
	30°: *50%* vs. *75%* F(1, 104) = 184.04, p < 0.001
	45°: *25%* vs. *50%* F(1, 104) = 142.13, p < 0.001
	45°: *25%* vs. *75%* F(1, 104) = 281.39, p < 0.001
	45°: *50%* vs. *75%* F(1, 104) = 106.80, p < 0.001
**Extensor digitorum longus**	
Factor 1	F(4, 52) = 8.33, p < 0.001
	Post-hoc analysis for Factor 1
	0° vs. 45°: F(1, 52) = 16.83, p = 0.012
	15° vs. 45°: F(1, 52) = 16.55, p = 0.013
Factor 2	F(2, 26) = 309.55, p < 0.001
	Post-hoc analysis for Factor 2
	25% vs. 50%: F(1, 26) = 247.56, p < 0.001
	25% vs. 75%: F(1, 26) = 392.80, p < 0.001
	25% vs. 50%: F(1, 26) = 200.69, p < 0.001
Interaction	F(8, 104) = 1.95, p = 0.060
**Gastrocnemius medialis**	
Factor 1	F(4, 44) = 4.47, p = 0.004
Factor 2	F(2, 22) = 13.90, p < 0.001
	Post-hoc analysis for Factor 2
	25% vs. 50%: F(1, 22) = 17.04, p = 0.005
	25% vs. 75%: F(1, 22) = 14.20, p = 0.009
	50% vs. 75%: F(1, 22) = 12.89, p = 0.013
Interaction	F(8, 88) = 4.45, p < 0.001
	Post-hoc for Factor 2 with Factor 1
	-15°: *25%* vs. *75%* F(1, 88) = 10.96, p = 0.021
	-15°: *50%* vs. *75%* F(1, 88) = 10.64, p = 0.023
	0°: *25%* vs. *50%* F(1, 88) = 11.94, p = 0.016
	0°: *25%* vs. *75%* F(1, 88) = 11.80, p = 0.017
	0°: *50%* vs. *75%* F(1, 88) = 10.24, p = 0.025
	15°: *25%* vs. *50%* F(1, 88) = 13.71, p = 0.010
	15°: *25%* vs. *75%* F(1, 88) = 12.62, p = 0.014
	15°: *50%* vs. *75%* F(1, 88) = 11.41, p = 0.018
	30°: *25%* vs. *50%* F(1, 88) = 13.14, p = 0.012
	30°: *25%* vs. *75%* F(1, 88) = 11.96, p = 0.016
	30°: *50%* vs. *75%* F(1, 88) = 11.08, p = 0.020
	45°: *25%* vs. *50%* F(1, 88) = 17.76, p = 0.004
	45°: *25%* vs. *75%* F(1, 88) = 17.12, p = 0.005
	45°: *50%* vs. *75%* F(1, 88) = 13.95, p = 0.010

**Gastrocnemius lateralis**	
Factor 1	F(4, 44) = 4.27, p = 0.005<br>Post-hoc analysis for Factor 1<br>0° vs. 30°: F(1, 44) = 15.00, p = 0.026
Factor 2	F(2, 22) = 22.47, p < 0.001<br>Post-hoc analysis for Factor 2<br>25% vs. 50%: F(1, 22) = 23.88, p = 0.001<br>25% vs. 75%: F(1, 22) = 22.62, p = 0.002<br>50% vs. 75%: F(1, 22) = 21.76, p = 0.002
Interaction	F(8, 88) = 2.84, p = 0.007<br>Post-hoc for Factor 1 with Factor 2<br>*50%*: 15° vs 30°: F(1, 88) = 18.77, p = 0.012<br>*50%*: 15° vs 45°: F(1, 88) = 12.81, p = 0.043<br>Post-hoc for Factor 2 with Factor 1<br>-15°: *25%* vs. *50%* F(1, 88) = 13.26, p = 0.012<br>-15°: *25%* vs. *75%* F(1, 88) = 20.99, p = 0.002<br>-15°: *50%* vs. *75%* F(1, 88) = 20.22, p = 0.003<br>0°: *25%* vs. *75%* F(1, 88) = 17.75, p = 0.004<br>0°: *50%* vs. *75%* F(1, 88) = 30.04, p = 0.001<br>15°: *25%* vs. *50%* F(1, 88) = 36.62, p < 0.001<br>15°: *25%* vs. *75%* F(1, 88) = 11.83, p = 0.017<br>15°: *50%* vs. *75%* F(1, 88) = 8.84, p = 0.038<br>30°: *25%* vs. *50%* F(1, 88) = 35.89, p < 0.001<br>30°: *25%* vs. *75%* F(1, 88) = 21.48, p = 0.002<br>30°: *50%* vs. *75%* F(1, 88) = 15.26, p = 0.007<br>45°: *25%* vs. *50%* F(1, 88) = 27.10, p = 0.001<br>45°: *25%* vs. *75%* F(1, 88) = 23.24, p = 0.002<br>45°: *50%* vs. *75%* F(1, 88) = 19.17, p = 0.003
**Soleus**	
Factor 1	F(4, 40) = 14.44, p < 0.001<br>Post-hoc analysis for Factor 1<br>-15° vs. 15°: F(1, 40) = 19.82, p = 0.012<br>-15° vs. 30°: F(1, 40) = 36.71, p = 0.001<br>-15° vs. 45°: F(1, 40) = 15.31, p = 0.029<br>0° vs. 15°: F(1, 40) = 33.21, p = 0.002<br>0° vs. 30°: F(1, 40) = 49.79, p < 0.001<br>0° vs. 45°: F(1, 40) = 13.25, p = 0.045<br>15° vs. 30°: F(1, 40) = 34.36, p = 0.002
Factor 2	F(2, 20) = 62.09, p < 0.001<br>Post-hoc analysis for Factor 2<br>25% vs. 50%: F(1, 20) = 51.85, p < 0.001<br>25% vs. 75%: F(1, 20) = 64.54, p < 0.001<br>50% vs. 75%: F(1, 20) = 59.93, p < 0.001
Interaction	F(8, 80) = 9.13, p < 0.001<br>Post-hoc for Factor 1 with Factor 2<br>*50%*: -15° vs 30°: F(1, 80) = 17.48, p = 0.019<br>*50%*: -15° vs 45°: F(1, 80) = 16.41, p = 0.023<br>*50%*: 0° vs 15°: F(1, 80) = 20.10, p = 0.012<br>*50%*: 0° vs 30°: F(1, 80) = 16.25, p = 0.024<br>*50%*: 0° vs 45°: F(1, 80) = 14.36, p = 0.035

	75%: -15° vs 15°: $F(1, 80) = 21.04$, $p = 0.010$<br>*75%*: -15° vs 30°: $F(1, 80) = 42.90$, $p = 0.001$<br>*75%*: -15° vs 45°: $F(1, 80) = 15.87$, $p = 0.026$<br>*75%*: 0° vs 15°: $F(1, 80) = 17.21$, $p = 0.020$<br>*75%*: 0° vs 30°: $F(1, 80) = 47.44$, $p < 0.001$<br>*75%*: 15° vs 30°: $F(1, 80) = 35.83$, $p = 0.001$<br>Post-hoc for Factor 2 with Factor 1<br>-15°: *25%* vs. *50%* $F(1, 80) = 16.35$, $p = 0.007$<br>-15°: *25%* vs. *75%* $F(1, 80) = 31.39$, $p = 0.001$<br>-15°: *50%* vs. *75%* $F(1, 80) = 27.95$, $p = 0.001$<br>0°: *25%* vs. *50%* $F(1, 80) = 30.71$, $p = 0.001$<br>0°: *25%* vs. *75%* $F(1, 80) = 51.96$, $p < 0.001$<br>0°: *50%* vs. *75%* $F(1, 80) = 53.19$, $p < 0.001$<br>15°: *25%* vs. *50%* $F(1, 80) = 41.46$, $p < 0.001$<br>15°: *25%* vs. *75%* $F(1, 80) = 54.40$, $p < 0.001$<br>15°: *50%* vs. *75%* $F(1, 80) = 46.54$, $p < 0.001$<br>30°: *25%* vs. *50%* $F(1, 80) = 24.47$, $p = 0.002$<br>30°: *25%* vs. *75%* $F(1, 80) = 62.60$, $p < 0.001$<br>30°: *50%* vs. *75%* $F(1, 80) = 56.29$, $p < 0.001$<br>45°: *25%* vs. *50%* $F(1, 80) = 29.61$, $p = 0.001$<br>45°: *25%* vs. *75%* $F(1, 80) = 38.61$, $p < 0.001$<br>45°: *50%* vs. *75%* $F(1, 80) = 35.67$, $p < 0.001$
**Peroneus longus**	
Factor 1	$F(4, 40) = 13.09$, $p < 0.001$<br>Post-hoc analysis for Factor 1<br>-15° vs. 30°: $F(1, 40) = 20.25$, $p = 0.011$<br>0° vs. 15°: $F(1, 40) = 14.53$, $p = 0.034$<br>0° vs. 30°: $F(1, 40) = 38.35$, $p = 0.001$<br>15° vs. 30°: $F(1, 40) = 23.46$, $p = 0.007$
Factor 2	$F(2, 20) = 17.00$, $p < 0.001$<br>Post-hoc analysis for Factor 2<br>25% vs. 50%: $F(1, 20) = 23.03$, $p = 0.002$<br>25% vs. 75%: $F(1, 20) = 17.45$, $p = 0.006$<br>50% vs. 75%: $F(1, 20) = 15.18$, $p = 0.009$
Interaction	$F(8, 80) = 4.66$, $p < 0.001$<br>Post-hoc for Factor 1 with Factor 2<br>*50%*: -15° vs 30°: $F(1, 80) = 25.14$, $p = 0.005$<br>*50%*: -15° vs 45°: $F(1, 80) = 13.71$, $p = 0.041$<br>*50%*: 0° vs 30°: $F(1, 80) = 34.43$, $p = 0.002$<br>*50%*: 15° vs 30°: $F(1, 80) = 22.27$, $p = 0.008$<br>*75%*: 0° vs 30°: $F(1, 80) = 29.08$, $p = 0.003$<br>Post-hoc for Factor 2 with Factor 1<br>-15°: *25%* vs. *50%* $F(1, 80) = 8.33$, $p = 0.049$<br>0°: *25%* vs. *50%* $F(1, 80) = 19.39$, $p = 0.004$<br>0°: *25%* vs. *75%* $F(1, 80) = 24.03$, $p = 0.002$<br>0°: *50%* vs. *75%* $F(1, 80) = 24.75$, $p = 0.002$<br>15°: *25%* vs. *50%* $F(1, 80) = 26.38$, $p = 0.001$<br>15°: *25%* vs. *75%* $F(1, 80) = 16.96$, $p = 0.006$<br>15°: *50%* vs. *75%* $F(1, 80) = 14.55$, $p = 0.010$<br>30°: *25%* vs. *50%* $F(1, 80) = 20.63$, $p = 0.003$<br>30°: *25%* vs. *75%* $F(1, 80) = 26.38$, $p = 0.001$

	30°: *50%* vs. *75%* F(1, 80) = 27.18, p = 0.001<br>45°: *25%* vs. *50%* F(1, 80) = 24.27, p = 0.002<br>45°: *25%* vs. *75%* F(1, 80) = 19.62, p = 0.004<br>45°: *50%* vs. *75%* F(1, 80) = 13.93, p = 0.012
