# Supplementary material for: In vivo tibialis anterior muscle mechanics through force estimation using ankle joint moment and shear wave elastography
Source: Sci Rep. 2025 Sep 12;15:32461. doi: 10.1038/s41598-025-18292-4 (PMC12432256; doi:10.1038/s41598-025-18292-4)
Supplement: Supplementary file 3 — Supplementary Information 3. [file 41598_2025_18292_MOESM3_ESM.pdf]

In vivo tibialis anterior muscle mechanics through force estimation using ankle joint moment and shear wave elastography

Supplementary 3: Saturation analysis of shear wave elastography data

The maximum measurable shear wave velocity (SWV) for the ultrasound system used in this study was 14.1 m/s (equivalent to 200 kPa). Across all ankle angles, the total shear modulus values recorded during maximal voluntary contractions (MVCs) remained well below this upper limit. For instance, the average shear modulus across MVCs was 122.96 ± 9.87 kPa, with a maximum value of 139.76 ± 24.91 kPa observed at 45° of plantarflexion (PF). During submaximal ramp contractions, the highest value recorded occurred at 75% MVC and 30° PF, reaching 153.93 ± 23.63 kPa.

These values represent the mean across valid pixels within the region of interest. In cases of substantial saturation, we would expect the average shear modulus of the remaining pixels to approach the system's measurement ceiling (i.e., ~200 kPa), as only the highest values are excluded. The fact that the observed mean values remain well below this threshold suggests that the degree of saturation is limited and that the underlying tissue does not exhibit extreme heterogeneity in tissue stiffness.

Nonetheless, due to inherent technical constraints in shear wave elastography, it remains challenging to fully quantify or exclude the influence of saturation. To account for this, we quantified the percentage of pixels reaching the maximum measurable SWV of 14.1 m/s within the region of interest, using this as a proxy of potential signal saturation.

> During MVC, the mean percentage of pixels reaching the maximum shear modulus was 20.97 ± 18.61%, with a median of 16.79%. Out of 210 trials collected during MVC, 62 (29.5%) exhibited at least 25% of pixels within the region of interest reaching the maximum shear modulus, while only 12 trials (5.7%) exceeded 50%, and just one trial exceeded 75%.
>
> For the submaximal ramp contractions, for 25%, 50%, and 75% MVC, the percentages of pixels reaching the maximum shear modulus value were 2.31 ± 4.47%, 17.01 ± 15.48%, and 24.50 ± 18.48%, respectively. Out of 420 trials collected during ramp contractions, 108 (25.7%) exhibited at least 25% of pixels within the region of interest reaching the maximum shear modulus, while only 13 trials (3.1%) exceeded 50%, and just two trials exceeded 75%.

Effect of Ankle Angle and Contraction Intensity on Pixel Saturation

To determine whether saturation varied with ankle angle and whether this may have impacted our conclusions about angle-dependent changes in shear modulus, the percentage of saturated pixels was analyzed per ankle angle (Table 1).

Table 1. Percentage of pixels reaching the maximum shear modulus, averaged across time frames, trial repetitions, and participants.

	Ankle angle				
	-15°	0°	15°	30°	45°
MVC	19.95 ± 12.64	15.96 ± 11.62	18.39 ± 13.15	20.10 ± 13.10	28.57 ± 15.60 $^{**, \square}$
25% MVC	0.45 ± 0.72	0.59 ± 0.76	1.81 ± 2.94	2.17 ± 2.73	6.70 ± 7.05
50% MVC	6.19 ± 4.50	12.88 ± 8.39 *	14.85 ± 11.87	23.39 ± 13.96 *	29.13 ± 17.06 $^{*, **, \circ}$
75% MVC	13.62 ± 6.53	18.42 ± 10.54	22.97 ± 15.56	35.05 ± 18.86 $^{*, **}$	34.36 ± 21.15 *

Values are mean ± standard deviation. *, **, $^{\circ}$, and $^{\square}$ indicate significant differences from the values measured at -15°, 0°, 15°, and 30° ankle angles, respectively. $p < 0.05$ for all pairwise comparisons.

During MVC, the percentage of pixels reached the maximum shear modulus value was significantly affected by the ankle angle ($F(4, 52) = 2.90$, $p = 0.031$). Post hoc comparisons revealed significant differences between 0° and 45° ($p = 0.018$), as well as between 30° and 45° ($p = 0.017$) (Table 1). The highest proportion was observed at 45° (28.57 ± 15.60 %), while the lowest was recorded at 0° (15.96 ± 11.62 %). This suggests a potential underestimation of shear modulus at 45°, which might otherwise show an apparent increase. Sasaki et al.[1], who studied a similar population over a narrower range of motion (-15° to 25°), reported a linear increase in total shear modulus with ankle angle, attributing it to the nonlinear elastic properties of connective tissues. Importantly, within the -15° to 30° range, where our data and Sasaki et al.[1] overlap, the observed saturation levels were comparable, making it unlikely that the absence of angle-dependent change in shear modulus was caused by saturation artifacts.

For the submaximal ramp contractions, the percentages of pixels reaching the maximum shear modulus value were significantly affected by ankle angle ($F(4, 52) = 17.04$, $p < 0.001$), contraction intensity ($F(2, 26) = 44.58$, $p < 0.001$), and their interaction ($F(8, 104) = 5.26$, $p < 0.001$). At 25% MVC, saturation was minimal and unaffected by ankle angle. At 50% MVC, significant differences were observed between -15° and 0°/30°/45°, 0° and 45°, and 15° and 45° ($p \leq 0.026$ for all). At 75% MVC, significant differences occurred between -15° and 30°/45°, and 0° and 30° ($p \leq 0.022$ for all) (Table 1). Saturation was consistently higher at greater contraction intensities. Still, at each intensity level, ankle angle continued to significantly affect shear modulus. Between 50% and 75% MVC, no saturation differences were found at most ankle angles, except at -15° ($p = 0.025$), suggesting saturation may have influenced results at higher intensities, particularly in more plantarflexed positions.

Impact of Saturation Thresholding on Main Outcomes

We also evaluated how applying saturation thresholds of 10% and 25% influenced the primary findings by excluding trials that exceeded each threshold (Figure 1).

(i) **MVC:** With a 25% saturation threshold, the previously non-significant effect of ankle angle on total shear modulus ($F(4, 52) = 2.33$, $p = 0.068$) remained non-significant ($F(4, 40) = 1.36$, $p = 0.266$; $n = 11$).

(ii) **Ramp contractions:** The effects of ankle angle ($F(4, 52) = 19.84$, $p < 0.001$), contraction intensity ($F(2, 26) = 43.91$, $p < 0.001$), and their interaction ($F(8, 104) = 3.25$, $p = 0.002$) remained significant. These results were replicated under the 25% saturation threshold (angle; $F(4, 16) = 5.10$, $p = 0.008$; intensity: $F(2, 8) = 7.49$, $p = 0.015$; interaction: $F(8, 32) = 3.17$, $p = 0.009$; $n = 5$), further supporting the robustness of the original findings.

As expected, the number of significant pairwise comparisons was reduced under thresholded conditions, likely due to decreased sensitivity from reduced trial counts and saturation-related signal underestimation. At the stricter 10% threshold, the number of valid trials was insufficient to support meaningful ANOVA analyses. Nevertheless, the overall pattern across ankle angles remained visually consistent (Figure 1).

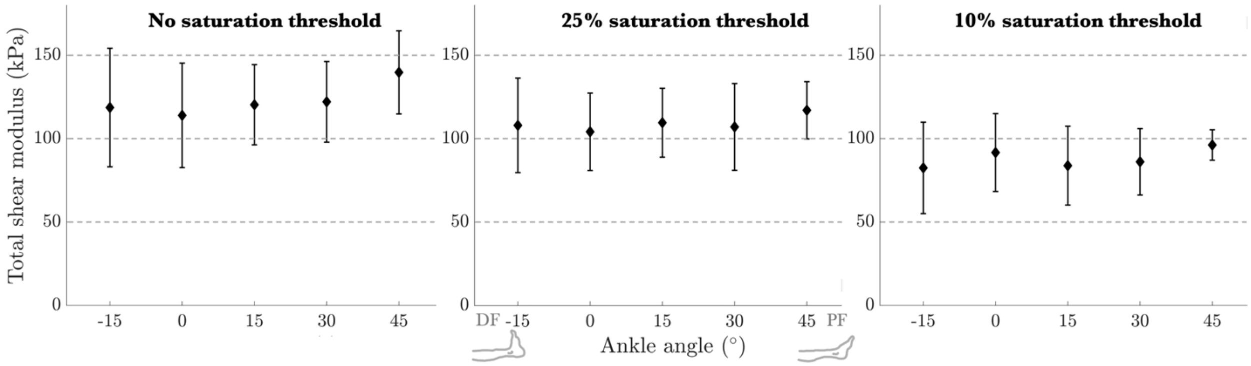

Figure 1. Total shear modulus of the tibialis anterior (TA) during maximum voluntary contraction (MVC) across the studied ankle angles. Data are shown for the full dataset (with no saturation threshold applied) and after applying 25% and 10% saturation thresholds. Error bars visualize standard deviations. DF: dorsiflexion, PF: plantar flexion.

References

1. Sasaki, K., Toyama, S. & Ishii, N. Length-force characteristics of in vivo human muscle reflected by supersonic shear imaging. *J Appl Physiol* **117**, 153–162 (2014).
